# Adjunctive host-directed therapies for pulmonary tuberculosis: a prospective, open-label, phase 2, randomised controlled trial

**DOI:** 10.1016/S2213-2600(20)30448-3

**Published:** 2021-08

**Authors:** Robert S Wallis, Sibuse Ginindza, Trevor Beattie, Nishanee Arjun, Morongwe Likoti, Vinodh A Edward, Mohammed Rassool, Khatija Ahmed, Katherine Fielding, Bintou A Ahidjo, Mboyo D T Vangu, Gavin Churchyard

**Affiliations:** a The Aurum Institute, Johannesburg, South Africa; b Department of Environmental Health Sciences, Yale School of Public Health, New Haven, CT, USA; c School of Pathology, University of the Witwatersrand, Johannesburg, South Africa; d Nuclear Medicine and Molecular Imaging, CM Johannesburg Academic Hospital, University of the Witwatersrand, Johannesburg, South Africa; e School of Public Health, University of the Witwatersrand, Johannesburg, South Africa; f Department of Internal Medicine, University of the Witwatersrand, Johannesburg, South Africa; g Clinical HIV Research Unit, Johannesburg, South Africa; h Setshaba Research Centre, Soshanguve, South Africa; i Department of Infectious Disease Epidemiology, London School of Hygiene & Tropical Medicine, London, UK; j Department of Medicine, Vanderbilt University, Nashville, TN, USA; k Department of Medicine, Case Western Reserve University, Cleveland, OH, USA; l Department of Medical Microbiology, Faculty of Health Science, University of Pretoria, Pretoria, South Africa

## Abstract

**Background:**

Current tuberculosis treatments leave patients with clinically significant lung injury and increased all-cause mortality post-cure. Adjunctive host-directed therapies could protect the lungs, improve long-term survival, and shorten treatment duration; however, few have been tested clinically. Therefore, we aimed to assess the safety and preliminary efficacy of four host-directed therapies for tuberculosis.

**Methods:**

In this prospective, open-label, phase 2, randomised controlled trial, patients with pulmonary tuberculosis were recruited at three clinical sites in South Africa. Eligible patients were aged 18–65 years, HIV-1-negative, and had rifampicin-susceptible *Mycobacterium tuberculosis*, a sputum Xpert cycle threshold of less than 20, and moderately advanced or far advanced disease on chest radiography. By use of numbers generated in blocks of ten and stratification by site, eligible patients were randomly assigned (1:1:1:1:1) to receive one of the four oral host-directed treatments plus standard tuberculosis treatment or standard treatment alone (the control group). Host-directed treatments were: CC-11050 (200 mg twice daily, taken with food; day 1–112); everolimus (0·5 mg/day; day 1–112); auranofin (3 mg/day for seven doses, then 6 mg/day; day 1–112); and ergocalciferol (5 mg on day 1, then 2·5 mg on day 28 and day 56). All study participants received oral rifabutin-substituted standard tuberculosis treatment for 180 days. Patients and clinicians were not masked to treatment assignment. Spirometry and sputum culture with solid and liquid media were done at baseline and up to 180 days at specified intervals throughout treatment. The primary endpoint was safety and tolerability up to day 210. Secondary preliminary efficacy endpoints were treatment effects on sputum microbiology (culture status at day 56 and the hazard ratio for stable culture conversion up to day 180) and lung function (FEV_1_ and forced vital capacity [FVC]) measured by spirometry at day 56, day 180, and day 540. Safety was analysed in the intention-to-treat population and preliminary efficacy primarily in the per-protocol population. The trial is registered at ClinicalTrials.gov, NCT02968927. Post-treatment follow-up was completed in 2020.

**Findings:**

Between Nov 18, 2016, and Sept 27, 2018, 200 patients were screened and randomly assigned to different treatment groups (n=40 per group, apart from n=39 in the everolimus group after one patient withdrew consent). 11 treatment-emergent serious adverse events occurred either during treatment or within 30 days after treatment discontinuation, of which three were attributable to a host-directed treatment. Life-threatening thrombocytopenia occurred in an auranofin recipient; apparent intra-abdominal sepsis leading to death occurred in another auranofin recipient and was classified as a suspected unexpected serious adverse reaction. Tuberculous spondylitis occurred as an apparent paradoxical reaction in a patient receiving ergocalciferol. Two patients in the control group had life-threatening, treatment-attributable liver injury. No treatment-emergent, treatment-attributable serious adverse events occurred in patients receiving CC-11050 or everolimus. Mean FEV_1_ in the control group was 61·7% of predicted (95% CI 56·3–67·1) at baseline and 69·1% (62·3–75·8) at day 180. Patients treated with CC-11050 and everolimus had increased recovery of FEV_1_ at day 180 relative to the control group (mean difference from control group 6·30%, 95% CI 0·06–12·54; p=0·048; and 6·56%, 0·18–12·95; p=0·044, respectively), whereas auranofin and ergocalciferol recipients did not. None of the treatments had an effect on FVC during 180 days of follow-up or on measures of sputum culture status over the course of the study.

**Interpretation:**

CC-11050 and everolimus were safe and reasonably well tolerated as adjunctive therapies for tuberculosis, and analysis of preliminary efficacy suggests they might also enhance the recovery of FEV_1_, a key measure of lung function and predictor of all-cause mortality. Further studies of these candidates are warranted.

**Funding:**

The Bill & Melinda Gates Foundation and the South African Medical Research Council.

**Research in context**

**Evidence before this study**

FEV_1_ is an independent, generalisable predictor of all-cause mortality. Tuberculosis leaves most patients with permanent impairment of lung function, including of FEV_1_. There is growing recognition that long-term all-cause mortality risks are increased after tuberculosis, to a degree consistent with impairment of FEV_1_. We searched PubMed for articles published in English between database inception and Dec 15, 2019, using the search terms “tuberculosis”, “clinical trial”, and “spirometry”. The search did not identify any studies of interventions to protect the lung done more recently than the 1960s. Several clinical trials of vitamin D have been done in patients with tuberculosis. Only one found that vitamin D enhanced sputum culture conversion at week 8 compared with standard treatment alone; none examined lung function. Studies of the type 4 phosphodiesterase inhibitor CC-11050 in experimental mouse and rabbit models of tuberculosis found that it improved the resolution of lung pathology and the clearance of *Mycobacterium tuberculosis* infection. One previous study of a non-specific phosphodiesterase inhibitor (pentoxifylline) found no benefit on short-term or long-term outcomes but did not examine effects on lung function.

**Added value of this study**

This phase 2 study found that adjunctive treatment with CC-11050 or everolimus in adults with pulmonary tuberculosis and baseline predictors of poor outcome was safe and well tolerated, and might have resulted in improved recovery of FEV_1_. Interventions to enhance the recovery of FEV1 in patients with tuberculosis might help to reduce the increased long-term mortality risks observed after treatment.

**Implications of all the available evidence**

More studies of CC-11050 and everolimus in patients with tuberculosis are warranted to further investigate their effects on lung function and assess their impact on long-term health outcomes.

## Introduction

Tuberculosis is a leading cause of morbidity and mortality globally.^1^ Current treatments are inadequate because they require patients to closely adhere to multidrug regimens that are long, complex, and often poorly tolerated or ineffective. Even if cured, most patients are left with bronchiectasis and fibrosis, permanent conditions that impair lung function and contribute to increased long-term mortality.^2^, ^3^

There is growing interest in the potential of adjunctive host-directed therapies to improve treatment options for patients with tuberculosis. As modulators of the host immune response, these treatments have the potential to protect the lungs, improve long-term survival, and shorten treatment duration by reducing lung inflammation, improving lesional drug penetration, and inducing antimicrobial activity in phagocytic cells.^4^ Although the list of candidates is long, few have been tested clinically, and many questions remain as to how they might best be evaluated.

Therefore, we aimed to assess the safety and preliminary efficacy of four adjunctive host-directed therapies in patients with hard-to-treat tuberculosis: CC-11050, everolimus, auranofin, and vitamin D (ergocalciferol). CC-11050 is a type 4 phosphodiesterase inhibitor with anti-inflammatory properties and has shown efficacy in preclinical models of *Mycobacterium tuberculosis* infection.^5^, ^6^ Everolimus is an inhibitor of serine/threonine-protein kinase mTOR. mTOR inhibitors induce autophagy, a potential mechanism of cellular antimycobacterial activity;^7^ they might also reduce inflammation and prevent fibrosis.^8^, ^9^ Auranofin, an orally bioavailable anti-inflammatory gold salt, shows in-vitro antimicrobial activity against *M tuberculosis*.^10^ Auranofin accumulates in macrophages, but has not been tested for anti-tuberculosis activity in cell culture or animals. Vitamin D is essential for host defences against *M tuberculosis*.^11^ Although several clinical trials have shown vitamin D to have no effect on tuberculosis sputum culture conversion,^12^ little is known regarding its clinical anti-inflammatory effects.^13^

## Methods

### Study design and participants

In this prospective, open-label, phase 2, randomised controlled trial, patients with pulmonary tuberculosis were recruited at three clinical sites in Greater Johannesburg and Pretoria, South Africa: the Tembisa Clinical Research Centre, Tembisa; the Clinical HIV Research Unit, Johannesburg; and the Setshaba Research Centre, Soshanguve. Patients attending neighbouring public health clinics were asked to give permission for a review of laboratory records for possible study eligibility. Those with positive sputum Cepheid Xpert *M tuberculosis*/rifampin (Xpert MTB/RIF; Sunnyvale, CA, USA) test results were asked to consent for further screening to enter the study.

Patients were aged 18–65 years, with Xpert MTB/RIF sputum testing showing rifampicin-susceptible *M tuberculosis* and one or more probes with a cycle threshold less than 20. Cycle threshold indicates the number of cycles of DNA amplification required to result in detection of the target and is inversely related to the log of the target copy number. Patients also had moderately advanced or far advanced pulmonary tuberculosis on chest radiography (appendix pp 29–30), had a bodyweight of 40–90 kg, and were willing and able to provide informed written consent or witnessed oral consent in the case of illiteracy according to South African and international guidelines before commencing any study procedures. Patients were also eligible to enter the trial on the basis of equivalent non-molecular tuberculosis testing; however, none did so. Patients with a history of tuberculosis, evidence of HIV-1 infection, chronic hepatitis B virus infection, diabetes, or chemistry or haematology values outside of specified ranges, and those who required corticosteroids in the past 28 days or other prohibited medications, were excluded. Women of childbearing potential were required to have a highly effective, non-hormonal method of contraception because of concerns regarding the potential teratogenicity of everolimus and possible pharmacokinetic interactions between hormonal contraceptives and rifabutin. The study provided placement of a copper-containing intrauterine contraceptive device at no cost to such women wishing to participate.

The protocol received ethics approval from the University of Witwatersrand Human Research Ethics Committee (reference number 151112), the London School of Hygiene & Tropical Medicine (reference number 10645), the South African Medicines Control Council (reference number 20160506), is registered as study 4297 in the South African Human Research Electronic Application System, and can be found online.

### Randomisation and masking

By use of numbers computer-generated by the study statistician (SG) in blocks of ten and with stratification by site, patients were randomly assigned (1:1:1:1:1) to receive CC-11050, everolimus, auranofin, or ergocalciferol in addition to standard tuberculosis therapy, or standard tuberculosis therapy alone (control). At each study site, the individuals responsible for assigning patients to treatment groups were not involved in evaluating treatment outcomes. Envelopes containing allocation information were provided to each site by SG. Patients and clinicians were not masked to treatment assignment. However, microbiology and spirometry laboratory personnel and senior study leadership (RSW and GC) were masked to treatment assignment. Summaries of study progress prepared by SG that described outcomes by treatment group were available only to members of the study's data and safety monitoring committee.

### Procedures

Participants randomly allocated to adjunctive host-directed tuberculosis therapies received CC-11050 (Celgene; Summit, NJ, USA), everolimus (Novartis; Basel, Switzerland), auranofin (Astellas [Milan, Italy], and later from Sebela Pharmaceuticals [Roswell, GA, USA] after Astellas discontinued its production), or ergocalciferol (Lennon; Port Elizabeth, South Africa) orally. Adjunctive CC-11050, everolimus, or auranofin were given from day 1 to day 112 as follows: 200 mg of CC-11050 twice a day with food; 0·5 mg/day of everolimus; or 3 mg/day of auranofin for the first week, which was then increased to 6 mg/day. Ergocalciferol was given as a 5 mg dose on day 1, then 2·5 mg on days 28 and 56. CC-11050 was given at the same dose used by Celgene in two phase 2 studies in patients with cutaneous lupus erythematosus (NCT01300208) and erythema nodosum leprosum (NCT03807362). The dose of everolimus was selected as the lowest showing human biological activity.^14^ Auranofin was used at the dose approved for the treatment of rheumatoid arthritis.^15^ Ergocalciferol dose was intermediate relative to doses in other tuberculosis treatment trials.^12^ All patients received 180 days of oral standard tuberculosis treatment: 2 months of daily isoniazid (Winthrop; Bridgewater, MA, USA) plus pyridoxine (Portfolio; Johannesburg, South Africa), rifabutin (Pfizer; New York, NY, USA), pyrazinamide (Sanofi; Bridgewater, MA, USA), and ethambutol (Sandoz; Holzkirchen, Germany), followed by 4 months of daily isoniazid (plus pyridoxine) and rifabutin. 300 mg/day of rifabutin was substituted for rifampicin (2HRbZE/4HRb) because of the potentially deleterious pharmacokinetic interactions of everolimus and CC-11050 with rifampicin.

We did sputum cultures using solid media (Löwenstein–Jensen medium, MEDIA-MAGE; Johannesburg, South Africa) and liquid media (Mycobacteria Growth Indicator Tube, BD, Franklin Lakes, NJ, USA) on days 0, 1, 7, 14, 21, 28, 35, 42, 56, 84, 112, 140, and 180, with particular attention to culture status on day 56. Patients were repeatedly encouraged to produce adequate sputum specimens, including through video instructional materials. Sputum induction was not done. *M tuberculosis* was identified by detection of immunogenic protein MPT64. Phenotypic drug susceptibility testing was done at baseline and on positive cultures after study day 112.

Spirometry was done at the study sites by use of EasyOne Pro instruments (NDD Medizintechnik, Zurich, Switzerland) beginning on study day 1. The protocol originally specified that spirometry be done on days 1, 14, 28, 56, 84, 112, 140, and 180, with particular attention to lung function on days 56 and 180. A protocol amendment later specified that spirometry should additionally be done on day 540. All sites used a single microbiology laboratory (the Tembisa Clinical Research Centre Biomedical Research Laboratory; Thembisa, South Africa) for *M tuberculosis* molecular testing, culture, and drug susceptibility testing, and a single commercial laboratory (Bio Analytical Research Corporation; Johannesburg, South Africa) for safety studies.

### Outcomes

The primary endpoint of the study was the safety and tolerability of the study treatments, in terms of treatment-emergent serious adverse events for CC-11050 and suspected unexpected serious adverse reactions for the other treatments up to day 210, and was centrally assessed. Secondary safety endpoints included treatment-emergent adverse events other than serious adverse events, categorised according to severity, drug-relatedness, and whether they lead to early withdrawal, and the proportion of patients with disease exacerbation (assessed at each scheduled visit) due to study treatments. Safety events were assessed by site personnel at each study visit and subsequently reviewed by the data and safety monitoring committee. Clinicians at each site provided initial assessments of severity (using adverse event grading tables from the Division of Allergy and Infectious Diseases) and treatment-relatedness. These assessments could be upgraded following central review.

Secondary preliminary efficacy endpoints were key measures of the effects of host-directed therapies on the clearance of *M tuberculosis* (as indicated by sputum culture status) and on lung function. The proportion of patients with positive sputum culture status was assessed at day 56 using solid culture medium, because previous trial data suggest culture status at this timepoint is a predictor of tuberculosis recurrence.^16^, ^17^ We also assessed culture status at several timepoints up to day 180 using solid and liquid medium. Treatment was considered unsuccessful if *M tuberculosis* was detected in more than one specimen after day 112. For the entire treatment period, stable culture conversion was defined as occurring on the day of the first of two consecutive sputum specimens obtained at least 2 weeks apart without growth of *M tuberculosis.* Specimens with liquid or solid cultures showing growth of *M tuberculosis* were considered positive. Instances in which patients were unable to provide a sputum specimen despite encouragement were considered negative. Cultures that were unevaluable because of contamination or other causes were considered missing. To assess lung function, FEV_1_ and forced vital capacity (FVC) were measured by spirometry, and change from control was assessed at numerous timepoints, with particular attention to days 56, 180, and 540. Spirometry findings were interpreted according to guidelines from the American Thoracic Society and the European Respiratory Society,^18^ with grade categories A–E considered acceptable. FEV_1_ was expressed as a percentage of the predicted value based on age, sex, height, and race (FEV_1_%).

Analyses of other secondary microbiology and lung function outcomes and of early biomarkers of treatment response, including changes in ^18^F-fluorodeoxyglucose-PET, CT imaging, and serum markers of inflammation, will be undertaken and reported separately.

### Statistical analysis

With 40 patients per group, this study was predicted to have a 77% power, at a two-sided 0·05 significance level, of detecting a significant difference in the proportion of serious adverse events between the control and experimental treatments, assuming proportions of 0·05 (n=2) in the control group and 0·3 (n=12) in the experimental groups. This sample size was considered appropriate to detect treatment-emergent serious adverse events attributable to CC-11050 in an exploratory trial in patients with tuberculosis because the sole previous phase 2 study (NCT01300208) included only 48 patients with cutaneous lupus erythematosus. We also considered microbiological outcomes in choosing sample size. Near-complete (≤1%) culture conversion at 2 months, measured by use of solid medium, has been proposed as a target for new 4-month regimens.^16^, ^17^ Poisson modelling indicates a 94% likelihood that a regimen meeting this target for the proportion of positive cultures would result in one or no patients with positive cultures out of 40. However, in the control group the expected proportion of positive cultures is 15–20%; therefore, the likelihood of yielding this result in the standard treatment group is only 0·3–1·5%. Data were insufficient to estimate statistical power for measures of lung function.

All safety endpoints were analysed in the intention-to-treat population, which comprised all randomly assigned patients who received at least one dose of a study drug. The modified intention-to-treat population included patients in the safety population, but excluded those wrongly enrolled (ie, not meeting enrolment criteria). The secondary efficacy outcomes for microbiology and lung function were analysed in the per-protocol population, which included patients in the modified intention-to-treat population, but excluded those who did not complete treatment or were found to have inadequate adherence (pill counts indicating that <299 [<83%] of 360 rifabutin 150 mg tablets were taken in total). The secondary efficacy analyses were repeated using the modified intention-to-treat population.

Safety data are presented descriptively, with particular attention to severity, treatment-relatedness, and action on study drug for serious adverse events and suspected unexpected serious adverse reactions. Logistic regression was used to assess associations of treatment group with culture-positive status measured after 56 days of treatment. We report unadjusted and adjusted odds ratios (ORs) and 95% CIs for culture status on solid medium, with the adjusted values controlling for baseline differences in time to positivity in automated liquid cultures. We individually compared experimental groups with the control group to calculate the hazard ratio (HR) for stable culture conversion up to 180 days using Cox proportional hazards regression. FEV_1_ and FVC at day 56, day 180, and day 540 were compared by ANCOVA. We did not adjust for multiple comparisons, but instead independently compared each candidate host-directed therapy with the control group, as each might be advanced independently on the basis of this analysis. Analyses were adjusted for baseline covariates. We considered adjusting for multiple baseline factors in each analysis, but found that the effect of additional factors was small. Missing data were excluded from analyses and not otherwise imputed or estimated.

Post-hoc analyses of FEV_1_ were done to investigate adjustment for study site in the per-protocol population and to examine the use of more stringent spirometry criteria at day 180 in the per-protocol population (grades A–D were considered acceptable). An additional post-hoc analysis fit a random effects model for the repeated measurements of FEV_1_% on a study day until day 180 using the modified intention-to-treat population and included an interaction between day and study group, and a random intercept for repeated measurement of FEV_1_%. Further post-hoc analyses separately examined treatment effects on culture conversion in liquid cultures in the per-protocol population using Cox proportional hazards methods.

All analyses were done by use of Stata (version 14). Study progress was monitored at regular intervals by an independent data and safety monitoring committee. This trial is registered as study 4297 in the South African Human Research Electronic Application System, as DOH-27–0616–5297 by the South African National Clinical Trial Registration, and as NCT02968927 by ClinicalTrials.gov.

### Role of the funding source

The Bill & Melinda Gates Foundation provided funding for this trial and contributed to the selection of host-directed therapy candidates and discussions of trial design. The South African Medical Research Council provided additional funding for administrative aspects of the study. Celgene provided CC-11050. The Aurum Institute served as a study sponsor. The funders of the study had no role in determining other aspects of study design, data collection, data analysis, data interpretation, or writing of the report.

## Results

**Figure 1:**
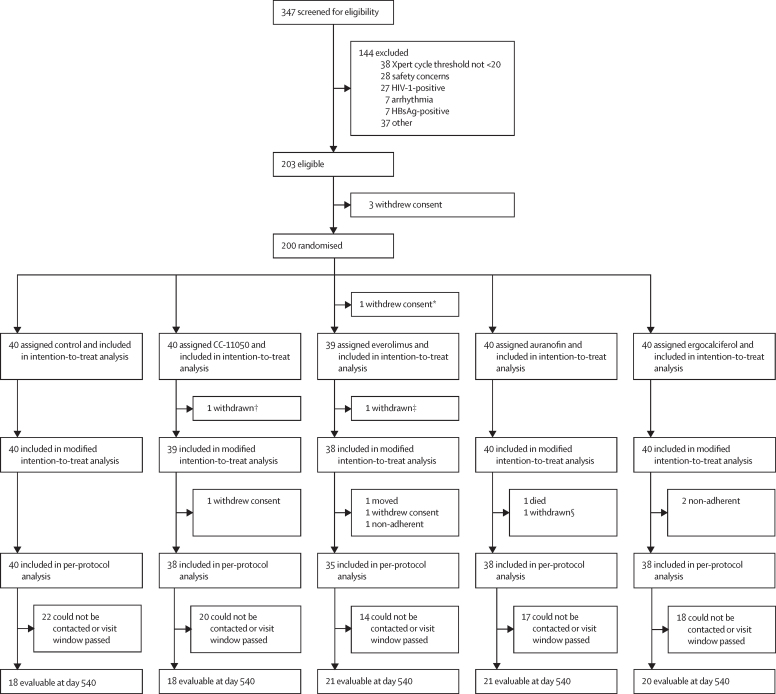
Trial profile *Withdrew consent before receiving any treatment. †Found to have spinal tuberculosis on baseline PET-CT scan. ‡Found to have elevated blood glucose at screening. §Required an excluded concomitant medication.

Between Nov 18, 2016, and Sept 27, 2018, we screened 347 patients with pulmonary tuberculosis for trial eligibility. Of these, 200 patients were enrolled and randomly assigned to different treatment groups (figure 1). The most frequent cause for trial exclusion was having an Xpert MTB/RIF cycle threshold of 20 or more, indicating insufficient sputum mycobacterial burden. The median age of the patients was 35 years (IQR 27–43) and they were predominantly male (table 1). Cavitary disease was present in 167 (84%) of 199 patients. Far advanced disease was present in 83 (42%) patients, most often due to having a total cavity diameter of 4 cm or more. The median time to detection in automated liquid culture was 103 h and the mean Xpert MTB/RIF cycle threshold was 16·8; both variables are inversely related to the sputum *M tuberculosis* burden (table 1). The mean FVC and the mean FEV_1_% indicated moderate lung impairment (table 1).

**Table 1:** Baseline characteristics of the intention-to-treat population

		**Control (n=40)**	**CC-11050 (n=40)**	**Everolimus (n=39)**	**Auranofin (n=40)**	**Ergocalciferol (n=40)**	**Total (n=199)**
Age, years		32 (26–43)	34 (27–41)	35 (26–43)	38 (30–43)	37 (29–49)	35 (27–43)
Weight, kg		54·7 (51·8–61·3)	54·5 (49·8–61·2)	55·2 (51·6–59·7)	53·3 (50·1–60·8)	53·8 (49·9–58·9)	54·4 (50·4–59·7)
Body-mass index, kg/m^2^		18·5 (16·8–19·9)	18·6 (17·0–19·6)	19·3 (17·1–21·5)	18·4 (17·2–20·0)	18·3 (16·5–20·0)	18·6 (17·0–20·3)
Sex							
	Female	2 (5%)	7 (18%)*	5 (13%)	3 (8%)*	7 (18%)*	24 (12%)
	Male	38 (95%)	33 (83%)*	34 (87%)	37 (93%)*	33 (83%)*	175 (88%)
Smoking history (former or current smoker)†		25 (63%)	24 (62%)	19 (50%)	20 (54%)	18 (46%)	106 (55%)
Total cavity diameter							
	0 cm	11 (28%)*	6 (15%)*	9 (23%)	2 (5%)	4 (10%)	32 (16%)
	>0 cm and <4 cm	24 (60%)*	23 (58%)*	20 (51%)	26 (65%)	28 (70%)	121 (61%)
	≥4 cm	5 (13%)*	11 (28%)*	10 (26%)	12 (30%)	8 (20%)	46 (23%)
Radiographic extent of disease							
	Moderately advanced	24 (60%)	23 (58%)*	27 (69%)	21 (53%)*	21 (53%)*	116 (58%)
	Far advanced	16 (40%)	17 (43%)*	12 (31%)	19 (48%)*	19 (48%)*	83 (42%)
Mean FEV1%‡		61·7 (56·3–67·1)	62·4 (55·2–69·5)	68·8 (62·9–74·8)	61·0 (54·6–67·4)	66·1 (58·0–74·2)	64·0 (61·1–66·9)
Mean FVC, L		3·12 (2·88–3·37)	3·00 (2·67–3·32)	3·08 (2·81–3·35)	3·07 (2·77–3·37)	3·04 (2·69–3·40)	3·06 (2·93–3·19)
Mean Xpert cycle threshold§		16·4 (15·4–17·4)	16·6 (15·6–17·7)	17·6 (16·2–19·1)	17·3 (16·2–18·4)	16·2 (15·0–17·4)	16·8 (16·3–17·4)
Time to positivity in mycobacterial growth indicator tube cultures, h		103 (84–140)	111 (100–135)	102 (88–144)	101 (91–127)	114 (87–132)	103 (89–134)

Data are median (IQR), n (%), or mean (95% CI). All participants were Black Africans. For more information on the definitions of radiographic disease extent, please see the appendix (pp 29–30). FEV1%=forced expiratory volume in 1 s as a proportion of predicted. FVC=forced vital capacity.

* Percentages do not add up to 100% because of rounding.

† Six patients had missing smoking history (n=1 for CC-11050, n=1 for everolimus, n=3 for auranofin, and n=1 for ergocalciferol). These patients are not included in the denominator for the calculation of percentages.

‡ One patient in the CC-11050 group had missing FEV1% data.

§ Two patients in the control group had missing Xpert cycle threshold data.

Three of 11 treatment-emergent serious adverse events were considered possibly or probably related to host-directed therapy (table 2). First, thrombocytopenia occurred 53 days after study entry in an auranofin recipient and resolved without consequence following auranofin discontinuation. Second, acute gastroenteritis, followed rapidly by hypotension, disseminated intravascular coagulation, and hypoxaemia arose in another auranofin recipient 2 weeks after study entry (table 2). The patient died despite broad-spectrum antimicrobials and intensive medical support. Blood cultures did not reveal a pathogen and autopsy was not done. The event was attributed to intra-abdominal sepsis and was classified as a suspected unexpected serious adverse reaction. Although there was an episode of acute hepatitis B virus infection in an auranofin recipient, it was thought to be unrelated to auranofin treatment on the basis of its time of onset (1 month after auranofin treatment had ended) and serological findings (conversion to HBsAg positivity accompanied by anti-HBc IgM positivity). Finally, tuberculous spondylitis arose 7 weeks after study entry in a patient who received ergocalciferol. This event was considered a paradoxical treatment reaction (localised disease exacerbation despite overall microbiological improvement) possibly due to ergocalciferol and resolved with symptomatic treatment. No patients in the CC-11050 group had treatment-emergent serious adverse events, nor were any treatment-emergent serious adverse events or suspected unexpected serious adverse reactions attributed to everolimus. A list of all adverse events, regardless of treatment-relatedness or seriousness, can be found in the appendix (p 2). No patients had treatments reduced or discontinued because of tolerability concerns.

**Table 2:** Treatment-emergent serious adverse events according to study group up to day 210 in the intention-to-treat (safety) population (n=199)

		**Seriousness**	**Days from study entry to onset**	**Treatment relatedness**		**Action on study medications**	**Adverse event outcome**
				To standard therapy	To host-directed therapy		
Control							
	Ischiorectal abscess	Required hospital admission	107	Unrelated	NA	Maintained	Resolved
	Drug-induced liver injury	Life-threatening	28	Probable	NA	Discontinued	Resolved
	Drug-induced liver injury	Life-threatening	37	Probable	NA	Interrupted	Resolved
CC-11050		..	..	..	..	..	..
Everolimus							
	Psychosis	Required hospital admission	124	Unrelated	Unrelated	Maintained	Resolved
Auranofin							
	Syncope	Required hospital admission	12	Unrelated	Unrelated	Maintained	Resolved
	Acute hepatitis B virus infection	Life-threatening	148	Unrelated	Unrelated	Interrupted	Resolved
	Thrombocytopenia	Life-threatening	53	Unrelated	Probable	Discontinued	Resolved
	Sepsis, disseminated intravascular coagulation, respiratory failure	Required hospital admission; resulted in death	14	Unrelated	Suspected unexpected serious adverse reaction	Host-directed therapy discontinued	Unresolved
Ergocalciferol							
	Dysfunctional uterine bleeding	Required hospital admission	110	Unrelated	Unrelated	Maintained	Resolved
	Tuberculous spondylitis	Medically significant	49	Unrelated	Possible	Maintained	Resolved
	Acute pancreatitis	Required hospital admission	195	Unrelated	Unrelated	Occurred post-treatment	Resolved

NA=not applicable.

**Figure 2:**
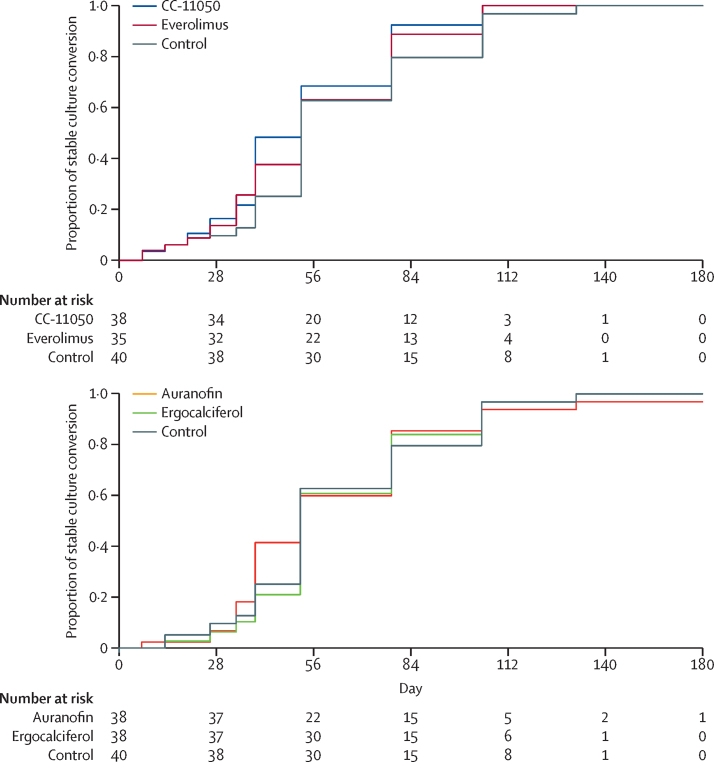
Proportion of patients with stable culture conversion with time by treatment group These data are based on combined results of solid and liquid cultures. The table indicates the numbers of patients at risk at key timepoints.

In the per-protocol population, compared with standard treatment alone, none of the four treatments had a significant effect on culture status at 56 days or on the hazard ratio for stable culture conversion up to day 180 (figure 2; Table 3, Table 4); this finding was confirmed in the modified intention-to-treat population (appendix p 23). Treatment succeeded in all but one patient, an auranofin recipient with positive cultures on solid medium on days 140 and 180.

**Table 3:** Culture status on solid medium after 56 days of treatment in the per-protocol population

	**Positive culture status**	**Unadjusted**		Adjusted*	
		OR (95% CI)	p value	OR (95% CI)	p value
Control	6/32 (19%)	NA	NA	NA	NA
CC-11050	6/32 (19%)	1·000 (0·285–3·509)	1·0	1·081 (0·301–3·820)	0·91
Everolimus	3/30 (10%)	0·482 (0·109–2·130)	0·34	0·490 (0·109–2·207)	0·35
Auranofin	4/32 (13%)	0·619 (0·157–2·444)	0·49	0·615 (0·153–2·472)	0·49
Ergocalciferol	5/31 (16%)	0·667 (0·168–2·642)	0·56	0·863 (0·230–3·245)	0·83

Data are n/N (%) or OR (95% CI), unless otherwise stated. Cultures that were unevaluable due to contamination or other causes were considered missing and were not otherwise imputed. NA=not applicable. OR=odds ratio for culture positivity relative to control.

* Adjusted for baseline differences in time to positivity in automated liquid cultures.

**Table 4:** HR for stable culture conversion up to day 180 in the per-protocol population (n=189)

	**Unadjusted**		Adjusted*	
	HR (95% CI)	p value	HR (95% CI)	p value
CC-11050	1·34 (0·86–2·10)	0·20	1·32 (0·84–2·07)	0·23
Everolimus	1·22 (0·77–1·93)	0·40	1·31 (0·83–2·09)	0·25
Auranofin	1·11 (0·71–1·74)	0·65	1·17 (0·74–1·84)	0·51
Ergocalciferol	1·02 (0·65–1·60)	0·93	0·96 (0·61–1·51)	0·87

Results include combined data for solid and liquid cultures. Separate analyses of liquid culture can be found in the appendix (p 24). Higher HR values for stable culture conversion indicate earlier conversion to negative status. HR=hazard ratio relative to control.

* Adjusted for baseline differences in time to positivity in automated liquid cultures.

**Figure 3:**
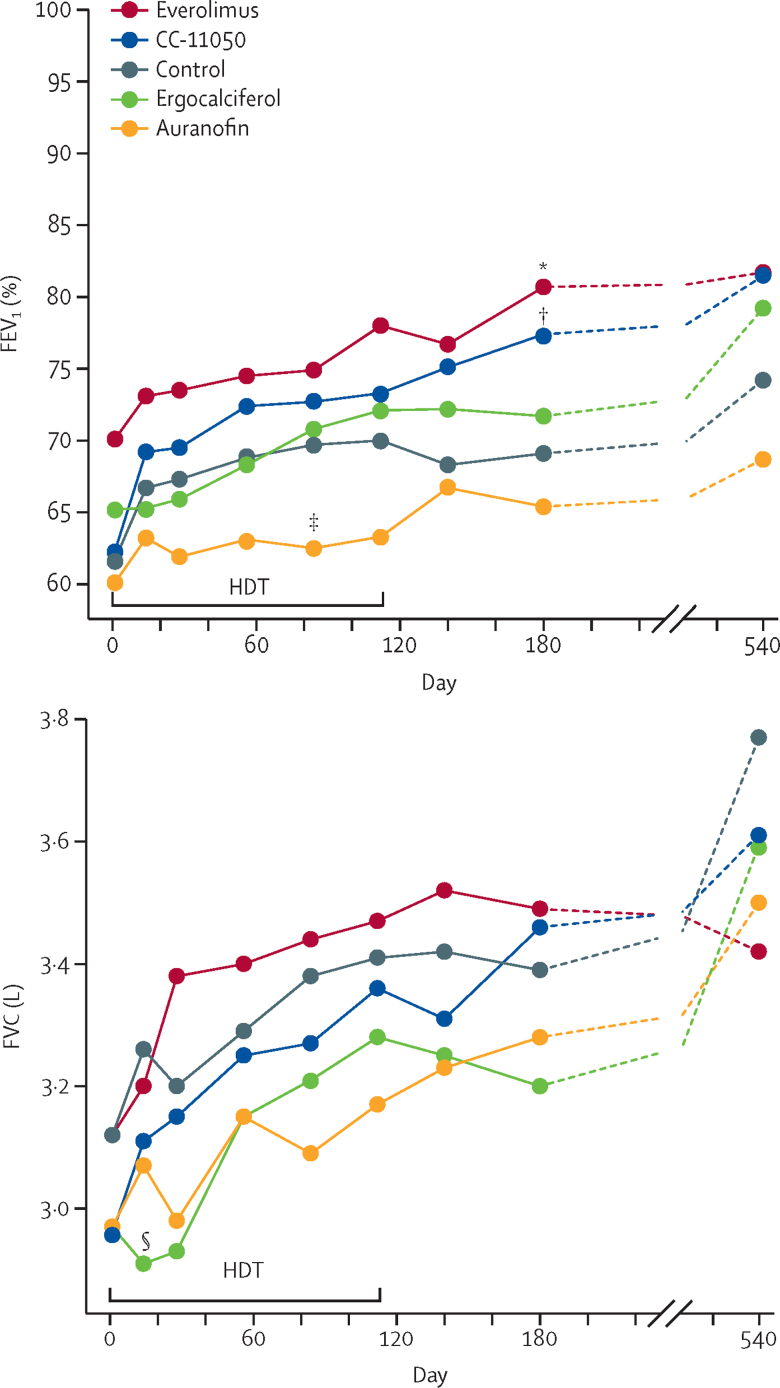
Lung function over time in the per-protocol population (A) Mean FEV_1_%. (B) Mean FVC. Please note the varying y-axes. HDTs were given until day 112, except for ergocalciferol, for which administration ceased on day 56. The symbols indicate timepoints that differ significantly from control after adjusting for differences at baseline. FEV_1_=forced expiratory volume in 1 s. FVC=forced vital capacity. HDT=host-directed therapy. *Everolimus at day 180. †CC-11050 at day 180. ‡Auranofin at day 84. §Ergocalciferol at day 14.

Mean values for FEV_1_% and FVC up to day 540 in the per-protocol population are shown in figure 3. The mean FEV_1_ for patients in the control group was 61·7% of predicted (95% CI 56·3–67·1) at baseline and 69·1% (62·3–75·8) at 180 days, with much of the apparent increase occurring early during standard treatment. All groups except for the ergocalciferol group showed an apparent increase in mean FEV_1_ from baseline to day 14, although none of these increases reached statistical significance (figure 3). None of the treatments affected FEV_1_ on day 56; however, mean FEV_1_ in the CC-11050 and everolimus groups did improve on day 180 (6·30, 95% CI 0·06–12·54, p=0·048, and 6·56, 95% CI 0·18–12·95, p=0·044, respectively; table 5); this finding was confirmed in the modified intention-to-treat population (appendix p 25). Auranofin transiently showed a detrimental treatment effect on FEV_1_ on day 84 (mean difference −6·44%, 95% CI −12·50 to −0·37; p=0·038; figure 3). 98 patients were eligible for extended evaluation on day 540, as permitted by a protocol amendment. The treatment groups retained the same rank order on day 540 as on day 180 with respect to FEV_1_ (figure 3), although sample sizes on day 540 were insufficient to maintain statistical significance. The mean FVC in the control group was 3·12 L (95% CI 2·88–3·37) at baseline and 3·39 L (3·13–3·66) at 180 days. Ergocalciferol transiently showed a detrimental effect on FVC on day 14 (mean difference −0·27 L, 95% CI −0·51 to −0·03; p=0·029), but none of the treatments had a lasting effect on FVC (figure 3; table 5).

**Table 5:** Spirometry outcomes in the per-protocol population (n=189)

	**Day 56 (n=189)**				**Day 180 (n=186)**				**Day 540 (n=98)**			
	Unadjusted		Adjusted		Unadjusted		Adjusted		Unadjusted		Adjusted	
	Mean (95% CI)	p value	Mean (95% CI)	p value	Mean (95% CI)	p value	Mean (95% CI)	p value	Mean (95% CI)	p value	Mean (95% CI)	p value
**FEV1%**												
CC-11050	3·54% (−5·57 to 12·66)	0·44	1·63% (−5·23 to 8·49)	0·64	8·40% (−0·24 to 17·04)	0·057	6·30% (0·06 to 12·54)	0·048	7·33% (−5·04 to 19·71)	0·24	0·58% (−9·09 to 10·25)	0·91
Everolimus	5·63% (−3·68 to 14·95)	0·23	0·40% (−6·62 to 7·42)	0·91	11·66% (2·84 to 20·49)	0·010	6·56% (0·18 to 12·95)	0·044	7·50% (−4·42 to 19·42)	0·22	1·92% (−7·36 to 11·20)	0·68
Auranofin	−6·17% (−15·28 to 2·95)	0·18	−5·14% (−11·96 to 1·68)	0·14	−3·70% (−12·40 to 5·00)	0·40	−2·79% (−9·04 to 3·45)	0·38	−5·45% (−17·38 to 6·47)	0·37	−5·44% (−14·62 to 3·74)	0·24
Ergocalciferol	−0·64% (−9·76 to 8·48)	0·89	−2·82% (−9·65 to 4·00)	0·42	2·35% (−6·34 to 11·05)	0·59	0·61% (−5·64 to 6·86)	0·85	5·03% (−7·03 to 17·10)	0·41	−0·52% (−9·91 to 8·87)	0·91
**FVC, L**												
CC-11050	−0·04 (−0·41 to 0·32)	0·82	0·07 (−0·18 to 0·32)	0·59	0·07 (−0·30 to 0·43)	0·71	0·18 (−0·09 to 0·45)	0·18	−0·16 (−0·69 to 0·38)	0·56	−0·09 (−0·52 to 0·34)	0·67
Everolimus	0·11 (−0·26 to 0·48)	0·57	0·11 (−0·14 to 0·36)	0·39	0·09 (−0·28 to 0·47)	0·62	0·11 (−0·17 to 0·38)	0·44	−0·35 (−0·87 to 0·16)	0·18	−0·12 (−0·54 to 0·29)	0·56
Auranofin	−0·14 (−0·50 to 0·23)	0·45	−0·04 (−0·28 to 0·21)	0·77	−0·12 (−0·49 to 0·25)	0·52	−0·02 (−0·29 to 0·25)	0·89	−0·27 (−0·79 to 0·25)	0·30	−0·11 (−0·53 to 0·31)	0·61
Ergocalciferol	−0·15 (−0·51 to 0·22)	0·43	−0·05 (−0·29 to 0·20)	0·72	−0·20 (−0·57 to 0·17)	0·29	−0·04 (−0·31 to 0·23)	0·78	−0·18 (−0·70 to 0·34)	0·49	−0·14 (−0·55 to 0·28)	0·52

Mean values indicate differences from the control group, before and after adjustment for differences from controls at baseline by ANCOVA. FEV1 was adjusted for baseline differences in FEV1. FVC was adjusted for baseline differences in FVC. Findings at 540 days are limited by reduced statistical power, as only 98 patients could be recalled for additional follow-up under a protocol amendment. FEV1%=forced expiratory volume in 1 s as a proportion of predicted. FVC=forced vital capacity.

Post-hoc analyses that separately examined culture conversion findings in liquid cultures (appendix p 24) confirmed the main study findings. Further post-hoc analyses of FEV_1_ at days 56 and 180 with study site as a factor for adjustment (appendix p 26) and at 180 days when using the more stringent criteria of spirometry grades of A–D (appendix p 27) also confirmed the main study findings. A random effects model was used to examine the repeated measurements of FEV_1_ according to study day; the analysis found a significant difference in slope parameter between the control and CC-11050 groups, and a difference approaching, but not reaching, significance for everolimus (appendix p 28).

## Discussion

This study examined the effects of four host-directed therapies for tuberculosis in a phase 2 clinical trial. To our knowledge, this trial is the first to specifically recruit hard-to-treat patients with tuberculosis at increased risk of poor outcomes based on initial chest radiography or sputum microbiology. In the era before chemotherapy, these factors were predictive of mortality.^19^ In the modern era, they predict relapse and permanent FEV_1_ loss.^2^, ^20^ To our knowledge, this randomised controlled trial is also the first study of tuberculosis treatment done since the 1960s to include spirometry as an endpoint.^21^, ^22^, ^23^ Three of the four candidates—CC-11050, everolimus, and auranofin—had not previously been studied in patients with tuberculosis, whereas vitamin D had been studied in several tuberculosis trials. Of these, we found CC-11050 and everolimus to be safe and reasonably well tolerated. The three treatment-emergent serious adverse events in this study occurred in recipients of auranofin (n=2) and ergocalciferol (n=1). Analyses of microbiological data showed no treatment effects on day 56 culture status or the HR for stable culture conversion.

In analyses of preliminary efficacy, both CC-11050 and everolimus appeared to enhance the recovery of FEV_1_ at 180 days; at 540 days, FEV_1_ results followed the same trajectory but sample sizes were insufficient to maintain statistical significance. Analyses of FEV_1_ in auranofin and ergocalciferol recipients and of FVC in all groups showed no difference compared with standard treatment alone. The magnitude of the observed effect on FEV_1_, approximately 6% for CC-11050 and everolimus at 180 days, corresponds to an absolute difference of 200 mL, twice the minimum effect considered clinically significant in chronic obstructive pulmonary disease (COPD).^24^ FEV_1_ can be impaired by the inability to fully inhale or rapidly exhale, which can be attributed to two predominant post-tuberculosis pathological mechanisms: fibrosis and bronchiectasis. The finding that enhanced recovery of FEV_1_ occurred relatively late (at day 180 but not day 56) during treatment is of particular interest. Inflammation due to recurring viral or bacterial lung infection is thought to drive progressive FEV_1_loss in COPD by promoting airway remodelling, a process that results in structural changes to the airways by depositing collagen in the extracellular matrix, altering the cellular composition of the epithelial barrier, and narrowing small airways.^25^, ^26^ The long-term effects of remodelling in COPD are increased airway obstruction and reduced lung compliance. Although little is known about airway remodelling in the post-tuberculosis lung, the delayed benefit of CC-11050 and everolimus could implicate effects on remodelling as a potential mechanism.

FEV_1_ is an independent, generalisable predictor of all-cause mortality, even in individuals with only mild to moderate impairment (ie, within the clinically normal range).^3^ In low-income countries where tuberculosis is most prevalent, standardised mortality risk doubles as FEV_1_ declines to 70% of predicted (moderately impaired), and doubles again as FEV_1_ further declines to 50% of predicted (severely impaired).^3^ Some of the patients enrolled in this study can therefore expect a four-times increase in long-term all-cause mortality because of permanent loss of FEV_1_. Multiple studies have confirmed that mortality risk is increased after tuberculosis.^27^ The excess deaths appear not to be due to recognised comorbidities such as HIV infection or diabetes, but instead reflect unexpected cardiovascular and respiratory illness.^27^, ^28^ Interventions to protect the lung and reduce lung inflammation might potentially offset as much as half of this excess mortality risk.

CC-11050, a type 4 phosphodiesterase inhibitor, was previously a backup compound for apremilast (ie, a replacement if apremilast failed to meet expectations), sharing similar chemical structures and biological activities. Apremilast is now approved for multiple anti-inflammatory indications. CC-11050 decreases the production of pro-inflammatory cytokines, including tumour necrosis factor, by preventing the degradation of cyclic AMP. In preclinical models of active tuberculosis in the mouse and rabbit, treatment with CC-11050 plus isoniazid reduced the number and size of lung lesions, ameliorated lung pathology, and reduced lung *M tuberculosis* colony-forming unit counts to a greater extent than did isoniazid alone.^5^, ^6^ In *M tuberculosis*-infected rabbits, CC-11050 additionally reduced the expression of multiple matrix metalloproteinase genes (particularly *MMP12*) and reduced the extracellular deposition of collagen in the lung.^6^ These effects might prevent lung fibrosis and post-inflammatory airway remodelling, thereby interrupting mechanisms responsible for permanent, progressive loss of lung function. CC-11050 has no direct anti-tuberculosis activity in vitro or when administered to animals alone. Its apparent antimicrobial effects when given with isoniazid in preclinical studies could be due to improved penetration or enhanced action of isoniazid in lung lesions. The absence of an effect of CC-11050 on the HR for stable culture conversion in this study might reflect the limitations of a small sample size. One previous study of a non-specific phosphodiesterase inhibitor (pentoxifylline) in patients with HIV and tuberculosis found no effects on sputum culture conversion or long-term outcomes, but did not examine lung function.^29^, ^30^

Everolimus, an inhibitor of mTOR, is used clinically at high doses for the treatment of some cancers, and at intermediate doses as an immunosuppressive agent for solid organ transplantation. Anti-inflammatory and anti-fibrotic effects of mTOR inhibitors have also been described.^8^, ^9^ At high concentrations, mTOR inhibitors promote autophagy in macrophages, activating a potential mechanism to restrict intracellular mycobacterial growth.^7^ The everolimus dose in this trial (0·5 mg/day) is the lowest showing human biological activity.^14^ Although FEV_1_ values at baseline in this study tended to be somewhat higher in everolimus recipients than in control recipients, the difference from controls at 180 days persisted after adjusting for this potential confounding factor. This clinical trial is, to our knowledge, the first of an mTOR inhibitor in tuberculosis.

Auranofin is an orally bioavailable gold salt introduced in the 1980s as a disease-modifying treatment for rheumatoid arthritis. In addition to their anti-inflammatory properties, gold salts show broad-spectrum antibacterial and antiviral activities in vitro, including against diverse intracellular pathogens such as *M tuberculosis*, HIV, and severe acute respiratory syndrome coronavirus 2.^10^, ^31^, ^32^ Gold becomes highly bound to macrophage and serum proteins in vivo,^33^ potentially interfering with delivery of active drug to the site of infection. In the late 1800s and early 1900s, sanocrysin, a double thiosulphate of gold(III) and sodium, was widely used for tuberculosis treatment.^34^ The use of sanocrysin was largely abandoned in 1931 when a careful clinical trial showed toxicity without clinical benefit.^35^ Auranofin appears to have reduced renal, pulmonary, and haematological toxicities compared with its parenteral predecessors, which were administered via intramuscular injection; however, gastrointestinal toxicities, particularly diarrhoea, remain common.^15^ Several cases of severe colitis due to gold therapy regardless of the route of administration have been reported; rare reports also describe colon perforation.^36^, ^37^ Although most cases occurred after many months of treatment, one report describes severe colitis occurring after 4 weeks of treatment with auranofin.^38^ For this reason, we were unable to exclude auranofin as a possible cause of death in one patient who had intra-abdominal sepsis, and classified the adverse event as a suspected unexpected serious adverse reaction. Additionally, another auranofin recipient in our study had life-threatening thrombocytopenia. Auranofin showed no clinical evidence of antimicrobial activity in this trial; indeed, the sole treatment failure occurred in an auranofin recipient. This imbalance of risk and benefit might influence future considerations of auranofin as a host-directed therapy for other infectious diseases.

Vitamin D is essential for host defences against tuberculosis.^11^ Vitamin D is required for the production of cathelicidin antimicrobial peptide in macrophages, and for the induction of autophagy following triggering of toll-like receptors.^39^ However, of eight randomised controlled trials of vitamin D in patients with pulmonary tuberculosis, only one showed a beneficial effect on sputum culture conversion.^12^ One of the trials described spinal disease arising during tuberculosis treatment in two vitamin D recipients,^40^ as it did in one patient in our study. Another trial reported one case of rapidly progressive fatal respiratory failure of undetermined cause within 2 weeks of initiation of vitamin D treatment.^41^ The underlying mechanism for these apparent paradoxical reactions is unknown, given the reported anti-inflammatory effects of vitamin D.^13^

The findings of this trial are best considered in the context of its experimental medicine design. The study's main shortcomings are a small sample size and low statistical power. Experimental groups were compared with the control without adjustment for multiple comparisons. Patients and clinicians could not readily be masked to treatment assignment because of the multiplicity of experimental treatments, which potentially introduced bias. As an exploratory study, its key findings will require verification in future trials. Further studies will be necessary to assess effects in patients with common comorbidities such as HIV-1 infection and diabetes, in patients with rifampicin-resistant disease, and in greater numbers of female patients. Repeated spirometry on day 2 in future studies would help to elucidate whether the apparent improvement in FEV_1_ on day 14 is a true early treatment response or merely a learned improvement in test taking. Larger studies with longer follow-up will be necessary to directly assess effects on mortality.

In summary, two adjunctive host-directed therapies for—CC-11050 and everolimus—showed adequate safety and tolerability in patients beginning treatment for pulmonary tuberculosis. An analysis of preliminary efficacy suggests that these therapies might preserve FEV_1_, a key measure of lung function and predictor of long-term all-cause mortality. Further definitive studies of CC-11050 and everolimus in patients with pulmonary tuberculosis are warranted.

For the **study protoco**l see https://bit.ly/2MA6bJL

## Data sharing

Data collected for the study, including deidentified individual participant data, a data dictionary defining each field in the set, and the statistical analysis plan will be made available to other researchers on request from the time of publication of this manuscript. Research proposals can be submitted to the corresponding author (RSW) at rwallis@auruminstitute.org.
