# Correction to *Lancet Respir Med* 2021; published online Feb 26. https://doi.org/10.1016/S2213-2600(20)30448-3

**DOI:** 10.1016/S2213-2600(21)00204-6

**Published:** 2021-06

*Wallis RS, Ginindza S, Beattie T, et al. Adjunctive host-directed therapies for pulmonary tuberculosis: a prospective, open-label, phase 2, randomised controlled trial.* Lancet Respir Med *2021; published online Feb 26. https://doi.org/10.1016/S2213-2600(20)30448-3*—In the author list, affiliations, and Contributors section of this Article, details for an additional author (Modulakgotla Sebe) have been added. The following affiliation for Trevor Beattie has also been added: “Department of Interdisciplinary Social Sciences, Utrecht University, Utrecht, Netherlands”. These corrections have been made to the online version as of April 19, 2021, and will be made to the printed version.

