# Supplementary material for: Adjunctive host-directed therapies for pulmonary tuberculosis: a prospective, open-label, phase 2, randomised controlled trial
Source: Lancet Respir Med. 2021 Aug;9(8):897–908. doi: 10.1016/S2213-2600(20)30448-3 (PMC8332197; doi:10.1016/S2213-2600(20)30448-3)
Supplement: Supplementary appendix [file mmc1.pdf]

THE LANCET
Respiratory Medicine

Supplementary appendix

This appendix formed part of the original submission and has been peer reviewed. We post it as supplied by the authors.

Supplement to: Wallis RS, Ginindza S, Beattie T, et al. Adjunctive host-directed therapies for pulmonary tuberculosis: a prospective, open-label, phase 2, randomised controlled trial. *Lancet Respir Med* 2021; published online March 16. http://dx.doi.org/10.1016/S2213-2600(20)30448-3.

TB-HDT Web Appendix

Table of contents

Table S1. Summary of Adverse Events by Maximum Severity and MedDRA Preferred Term 2

Table S2. Hazard ratio (HR) for SSC in the mITT population relative to control. 23

Table S3. Hazard ratio (HR) for SSC in the PP population relative to control, liquid cultures only. 24

Table S4. Spirometry outcomes in the mITT population. 25

Table S5. Spirometry outcomes in the PP population, including an adjustment for site. 26

Table S6. Effects on FEV1 at 6 months considering only ATS/ERS grade categories A-D. 27

Table S7. Effects on FEV1 over time in the mITT population using a random effects model. 28

Table S8. Criteria for classifying radiographic extent of disease in tuberculosis. 29

Table S1. Summary of Adverse Events by Maximum Severity and MedDRA Preferred Term

Intent-to-Treat (Safety) Population

Note: Patients are counted once for each preferred term and once for each system organ class (SOC) per the great est severity. SOCs are sorted in alphabetic order, and preferred terms are sorted in alphabetic order within SOCs.

**System Organ Class Preferred Term Severity**	**Control (N=40)**	**CC-11050 (N=40)**	**Everolimus (N=39)**	**Auranofin (N=40)**	**Vitamin D (N=40)**	**Total (N=199)**
**Any Event**	34 (85.0)	36 (90.0)	32 (82.1)	36 (90.0)	39 (97.5)	177 (88.9)
Mild	11 (27.5)	15 (37.5)	12 (30.8)	15 (37.5)	19 (47.5)	72 (36.2)
Moderate	19 (47.5)	19 (47.5)	19 (48.7)	17 (42.5)	16 (40.0)	90 (45.2)
Severe	4 (10.0)	2 (5.0)	1 (2.6)	4 (10.0)	4 (10.0)	15 (7.5)
**Blood and lymphatic system disorders**	2 (5.0)	10 (25.0)	6 (15.4)	4 (10.0)	5 (12.5)	27 (13.6)
Mild	2 (5.0)	6 (15.0)	3 (7.7)	3 (7.5)	3 (7.5)	17 (8.5)
Moderate	0	4 (10.0)	3 (7.7)	0	1 (2.5)	8 (4.0)
Severe	0	0	0	1 (2.5)	1 (2.5)	2 (1.0)
Anaemia	0	3 (7.5)	2 (5.1)	1 (2.5)	2 (5.0)	8 (4.0)
Mild	0	3 (7.5)	1 (2.6)	1 (2.5)	2 (5.0)	7 (3.5)
Moderate	0	0	1 (2.6)	0	0	1 (0.5)
Severe	0	0	0	0	0	0
Hypochromic anaemia	1 (2.5)	1 (2.5)	0	0	0	2 (1.0)
Mild	1 (2.5)	1 (2.5)	0	0	0	2 (1.0)
Moderate	0	0	0	0	0	0
Severe	0	0	0	0	0	0
Leukopenia	0	2 (5.0)	1 (2.6)	0	2 (5.0)	5 (2.5)
Mild	0	1 (2.5)	1 (2.6)	0	1 (2.5)	3 (1.5)
Moderate	0	1 (2.5)	0	0	1 (2.5)	2 (1.0)
Severe	0	0	0	0	0	0
Lymphadenopathy	0	1 (2.5)	1 (2.6)	0	0	2 (1.0)
Mild	0	1 (2.5)	1 (2.6)	0	0	2 (1.0)
Moderate	0	0	0	0	0	0
Severe	0	0	0	0	0	0
Lymphopenia	0	1 (2.5)	1 (2.6)	0	2 (5.0)	4 (2.0)
Mild	0	1 (2.5)	0	0	1 (2.5)	2 (1.0)
Moderate	0	0	1 (2.6)	0	0	1 (0.5)
Severe	0	0	0	0	1 (2.5)	1 (0.5)
Neutropenia	0	2 (5.0)	0	0	1 (2.5)	3 (1.5)
Mild	0	2 (5.0)	0	0	0	2 (1.0)
Moderate	0	0	0	0	1 (2.5)	1 (0.5)

System Organ Class Preferred Term Severity	Control (N=40)	CC-11050 (N=40)	Everolimus (N=39)	Auranofin (N=40)	Vitamin D (N=40)	Total (N=199)
Severe	0	0	0	0	0	0
Thrombocytopenia	0	1 (2.5)	0	1 (2.5)	1 (2.5)	3 (1.5)
Mild	0	1 (2.5)	0	0	0	1 (0.5)
Moderate	0	0	0	0	1 (2.5)	1 (0.5)
Severe	0	0	0	1 (2.5)	0	1 (0.5)
Thrombocytosis	0	2 (5.0)	1 (2.6)	2 (5.0)	0	5 (2.5)
Mild	0	0	0	2 (5.0)	0	2 (1.0)
Moderate	0	2 (5.0)	1 (2.6)	0	0	3 (1.5)
Severe	0	0	0	0	0	0
White blood cell disorder	1 (2.5)	1 (2.5)	0	0	0	2 (1.0)
Mild	1 (2.5)	0	0	0	0	1 (0.5)
Moderate	0	1 (2.5)	0	0	0	1 (0.5)
Severe	0	0	0	0	0	0
**Cardiac disorders**	4 (10.0)	5 (12.5)	1 (2.6)	3 (7.5)	5 (12.5)	18 (9.0)
Mild	4 (10.0)	5 (12.5)	1 (2.6)	3 (7.5)	5 (12.5)	18 (9.0)
Moderate	0	0	0	0	0	0
Severe	0	0	0	0	0	0
AV block first degree	0	2 (5.0)	0	0	0	2 (1.0)
Mild	0	2 (5.0)	0	0	0	2 (1.0)
Moderate	0	0	0	0	0	0
Severe	0	0	0	0	0	0
Bradycardia	2 (5.0)	2 (5.0)	1 (2.6)	2 (5.0)	2 (5.0)	9 (4.5)
Mild	2 (5.0)	2 (5.0)	1 (2.6)	2 (5.0)	2 (5.0)	9 (4.5)
Moderate	0	0	0	0	0	0
Severe	0	0	0	0	0	0
LVH	0	0	0	1 (2.5)	0	1 (0.5)
Mild	0	0	0	1 (2.5)	0	1 (0.5)
Moderate	0	0	0	0	0	0
Severe	0	0	0	0	0	0
Palpitations	1 (2.5)	0	0	0	0	1 (0.5)
Mild	1 (2.5)	0	0	0	0	1 (0.5)
Moderate	0	0	0	0	0	0
Severe	0	0	0	0	0	0
Tachycardia	1 (2.5)	2 (5.0)	0	0	3 (7.5)	6 (3.0)
Mild	1 (2.5)	2 (5.0)	0	0	3 (7.5)	6 (3.0)
Moderate	0	0	0	0	0	0
Severe	0	0	0	0	0	0
**Ear and labyrinth disorders**	0	0	1 (2.6)	1 (2.5)	0	2 (1.0)

System Organ Class Preferred Term Severity	Control (N=40)	CC-11050 (N=40)	Everolimus (N=39)	Auranofin (N=40)	Vitamin D (N=40)	Total (N=199)
Mild	0	0	1 (2.6)	1 (2.5)	0	2 (1.0)
Moderate	0	0	0	0	0	0
Severe	0	0	0	0	0	0
Ear congestion	0	0	1 (2.6)	0	0	1 (0.5)
Mild	0	0	1 (2.6)	0	0	1 (0.5)
Moderate	0	0	0	0	0	0
Severe	0	0	0	0	0	0
Ear pain	0	0	0	1 (2.5)	0	1 (0.5)
Mild	0	0	0	1 (2.5)	0	1 (0.5)
Moderate	0	0	0	0	0	0
Severe	0	0	0	0	0	0
Excessive cerumen production	0	0	1 (2.6)	0	0	1 (0.5)
Mild	0	0	1 (2.6)	0	0	1 (0.5)
Moderate	0	0	0	0	0	0
Severe	0	0	0	0	0	0
**Eye disorders**	1 (2.5)	0	0	2 (5.0)	0	3 (1.5)
Mild	1 (2.5)	0	0	2 (5.0)	0	3 (1.5)
Moderate	0	0	0	0	0	0
Severe	0	0	0	0	0	0
Eye pruritus	1 (2.5)	0	0	1 (2.5)	0	2 (1.0)
Mild	1 (2.5)	0	0	1 (2.5)	0	2 (1.0)
Moderate	0	0	0	0	0	0
Severe	0	0	0	0	0	0
Ocular hyperaemia	0	0	0	1 (2.5)	0	1 (0.5)
Mild	0	0	0	1 (2.5)	0	1 (0.5)
Moderate	0	0	0	0	0	0
Severe	0	0	0	0	0	0
**Gastrointestinal disorders**	7 (17.5)	4 (10.0)	9 (23.1)	14 (35.0)	8 (20.0)	42 (21.1)
Mild	4 (10.0)	1 (2.5)	3 (7.7)	10 (25.0)	5 (12.5)	23 (11.6)
Moderate	3 (7.5)	3 (7.5)	6 (15.4)	4 (10.0)	3 (7.5)	19 (9.5)
Severe	0	0	0	0	0	0
Abdominal discomfort	1 (2.5)	0	0	0	0	1 (0.5)
Mild	1 (2.5)	0	0	0	0	1 (0.5)
Moderate	0	0	0	0	0	0
Severe	0	0	0	0	0	0
Abdominal pain	2 (5.0)	0	3 (7.7)	2 (5.0)	2 (5.0)	9 (4.5)
Mild	2 (5.0)	0	3 (7.7)	2 (5.0)	1 (2.5)	8 (4.0)

System Organ<br>Class Preferred Term<br>Severity	Control (N=40)	CC-11050 (N=40)	Everolimus (N=39)	Auranofin (N=40)	Vitamin D (N=40)	Total (N=199)
Moderate	0	0	0	0	1 (2.5)	1 (0.5)
Severe	0	0	0	0	0	0
Abdominal pain lower	0	1 (2.5)	0	0	0	1 (0.5)
Mild	0	0	0	0	0	0
Moderate	0	1 (2.5)	0	0	0	1 (0.5)
Severe	0	0	0	0	0	0
Abdominal pain upper	1 (2.5)	0	0	1 (2.5)	0	2 (1.0)
Mild	0	0	0	1 (2.5)	0	1 (0.5)
Moderate	1 (2.5)	0	0	0	0	1 (0.5)
Severe	0	0	0	0	0	0
Abdominal tenderness	1 (2.5)	0	0	1 (2.5)	1 (2.5)	3 (1.5)
Mild	1 (2.5)	0	0	1 (2.5)	1 (2.5)	3 (1.5)
Moderate	0	0	0	0	0	0
Severe	0	0	0	0	0	0
Constipation	2 (5.0)	0	0	1 (2.5)	2 (5.0)	5 (2.5)
Mild	2 (5.0)	0	0	1 (2.5)	2 (5.0)	5 (2.5)
Moderate	0	0	0	0	0	0
Severe	0	0	0	0	0	0
Dental caries	0	1 (2.5)	1 (2.6)	0	0	2 (1.0)
Mild	0	0	0	0	0	0
Moderate	0	1 (2.5)	1 (2.6)	0	0	2 (1.0)
Severe	0	0	0	0	0	0
Diarrhoea	1 (2.5)	0	1 (2.6)	7 (17.5)	2 (5.0)	11 (5.5)
Mild	1 (2.5)	0	0	3 (7.5)	2 (5.0)	6 (3.0)
Moderate	0	0	1 (2.6)	4 (10.0)	0	5 (2.5)
Severe	0	0	0	0	0	0
Dyspepsia	1 (2.5)	0	0	0	0	1 (0.5)
Mild	1 (2.5)	0	0	0	0	1 (0.5)
Moderate	0	0	0	0	0	0
Severe	0	0	0	0	0	0
Gastritis	0	0	0	1 (2.5)	1 (2.5)	2 (1.0)
Mild	0	0	0	1 (2.5)	0	1 (0.5)
Moderate	0	0	0	0	1 (2.5)	1 (0.5)
Severe	0	0	0	0	0	0
GE reflux disease	0	0	0	1 (2.5)	0	1 (0.5)
Mild	0	0	0	1 (2.5)	0	1 (0.5)
Moderate	0	0	0	0	0	0
Severe	0	0	0	0	0	0

System Organ Class Preferred Term Severity	Control (N=40)	CC-11050 (N=40)	Everolimus (N=39)	Auranofin (N=40)	Vitamin D (N=40)	Total (N=199)
Haemorrhoids	1 (2.5)	0	0	1 (2.5)	0	2 (1.0)
Mild	0	0	0	1 (2.5)	0	1 (0.5)
Moderate	1 (2.5)	0	0	0	0	1 (0.5)
Severe	0	0	0	0	0	0
Hyperaesthesia teeth	1 (2.5)	0	0	0	0	1 (0.5)
Mild	1 (2.5)	0	0	0	0	1 (0.5)
Moderate	0	0	0	0	0	0
Severe	0	0	0	0	0	0
Nausea	2 (5.0)	1 (2.5)	0	1 (2.5)	0	4 (2.0)
Mild	2 (5.0)	0	0	0	0	2 (1.0)
Moderate	0	1 (2.5)	0	1 (2.5)	0	2 (1.0)
Severe	0	0	0	0	0	0
Pancreatitis acute	0	0	0	0	1 (2.5)	1 (0.5)
Mild	0	0	0	0	0	0
Moderate	0	0	0	0	1 (2.5)	1 (0.5)
Severe	0	0	0	0	0	0
Peptic ulcer	0	0	3 (7.7)	0	0	3 (1.5)
Mild	0	0	1 (2.6)	0	0	1 (0.5)
Moderate	0	0	2 (5.1)	0	0	2 (1.0)
Severe	0	0	0	0	0	0
Salivary hypersecretion	0	0	1 (2.6)	0	0	1 (0.5)
Mild	0	0	1 (2.6)	0	0	1 (0.5)
Moderate	0	0	0	0	0	0
Severe	0	0	0	0	0	0
Toothache	0	0	1 (2.6)	1 (2.5)	0	2 (1.0)
Mild	0	0	0	0	0	0
Moderate	0	0	1 (2.6)	1 (2.5)	0	2 (1.0)
Severe	0	0	0	0	0	0
Vomiting	2 (5.0)	1 (2.5)	2 (5.1)	4 (10.0)	3 (7.5)	12 (6.0)
Mild	1 (2.5)	1 (2.5)	1 (2.6)	3 (7.5)	3 (7.5)	9 (4.5)
Moderate	1 (2.5)	0	1 (2.6)	1 (2.5)	0	3 (1.5)
Severe	0	0	0	0	0	0
**General disorders and administration site conditions**	5 (12.5)	8 (20.0)	5 (12.8)	7 (17.5)	8 (20.0)	33 (16.6)
Mild	4 (10.0)	5 (12.5)	3 (7.7)	4 (10.0)	5 (12.5)	21 (10.6)
Moderate	1 (2.5)	3 (7.5)	2 (5.1)	3 (7.5)	3 (7.5)	12 (6.0)
Severe	0	0	0	0	0	0
Adverse drug reaction	0	0	0	0	1 (2.5)	1 (0.5)

System Organ<br>Class Preferred Term<br>Severity	Control (N=40)	CC-11050 (N=40)	Everolimus (N=39)	Auranofin (N=40)	Vitamin D (N=40)	Total (N=199)
Mild	0	0	0	0	1 (2.5)	1 (0.5)
Moderate	0	0	0	0	0	0
Severe	0	0	0	0	0	0
Adverse event	2 (5.0)	2 (5.0)	0	1 (2.5)	0	5 (2.5)
Mild	2 (5.0)	2 (5.0)	0	1 (2.5)	0	5 (2.5)
Moderate	0	0	0	0	0	0
Severe	0	0	0	0	0	0
Asthenia	0	2 (5.0)	1 (2.6)	1 (2.5)	1 (2.5)	5 (2.5)
Mild	0	2 (5.0)	1 (2.6)	0	1 (2.5)	4 (2.0)
Moderate	0	0	0	1 (2.5)	0	1 (0.5)
Severe	0	0	0	0	0	0
Chills	0	1 (2.5)	0	0	1 (2.5)	2 (1.0)
Mild	0	1 (2.5)	0	0	1 (2.5)	2 (1.0)
Moderate	0	0	0	0	0	0
Severe	0	0	0	0	0	0
Crepitations	0	0	0	1 (2.5)	0	1 (0.5)
Mild	0	0	0	1 (2.5)	0	1 (0.5)
Moderate	0	0	0	0	0	0
Severe	0	0	0	0	0	0
Fatigue	1 (2.5)	1 (2.5)	0	0	0	2 (1.0)
Mild	1 (2.5)	1 (2.5)	0	0	0	2 (1.0)
Moderate	0	0	0	0	0	0
Severe	0	0	0	0	0	0
Feeling cold	0	0	0	0	1 (2.5)	1 (0.5)
Mild	0	0	0	0	1 (2.5)	1 (0.5)
Moderate	0	0	0	0	0	0
Severe	0	0	0	0	0	0
Feeling hot	0	1 (2.5)	0	0	0	1 (0.5)
Mild	0	1 (2.5)	0	0	0	1 (0.5)
Moderate	0	0	0	0	0	0
Severe	0	0	0	0	0	0
Influenza like illness	0	0	0	0	1 (2.5)	1 (0.5)
Mild	0	0	0	0	1 (2.5)	1 (0.5)
Moderate	0	0	0	0	0	0
Severe	0	0	0	0	0	0
Injection site pain	0	0	0	0	2 (5.0)	2 (1.0)
Mild	0	0	0	0	2 (5.0)	2 (1.0)
Moderate	0	0	0	0	0	0

System Organ<br>Class Preferred Term<br>Severity	Control (N=40)	CC-11050 (N=40)	Everolimus (N=39)	Auranofin (N=40)	Vitamin D (N=40)	Total (N=199)
Severe	0	0	0	0	0	0
Oedema peripheral	0	1 (2.5)	0	0	0	1 (0.5)
Mild	0	0	0	0	0	0
Moderate	0	1 (2.5)	0	0	0	1 (0.5)
Severe	0	0	0	0	0	0
Pain	1 (2.5)	1 (2.5)	0	1 (2.5)	0	3 (1.5)
Mild	1 (2.5)	1 (2.5)	0	1 (2.5)	0	3 (1.5)
Moderate	0	0	0	0	0	0
Severe	0	0	0	0	0	0
Paradoxical drug reaction	0	3 (7.5)	3 (7.7)	2 (5.0)	3 (7.5)	11 (5.5)
Mild	0	1 (2.5)	1 (2.6)	0	0	2 (1.0)
Moderate	0	2 (5.0)	2 (5.1)	2 (5.0)	3 (7.5)	9 (4.5)
Severe	0	0	0	0	0	0
Peripheral swelling	0	1 (2.5)	0	0	0	1 (0.5)
Mild	0	1 (2.5)	0	0	0	1 (0.5)
Moderate	0	0	0	0	0	0
Severe	0	0	0	0	0	0
Pyrexia	1 (2.5)	1 (2.5)	1 (2.6)	0	0	3 (1.5)
Mild	0	1 (2.5)	1 (2.6)	0	0	2 (1.0)
Moderate	1 (2.5)	0	0	0	0	1 (0.5)
Severe	0	0	0	0	0	0
Swelling	0	0	0	1 (2.5)	0	1 (0.5)
Mild	0	0	0	1 (2.5)	0	1 (0.5)
Moderate	0	0	0	0	0	0
Severe	0	0	0	0	0	0
**Hepatobiliary disorders**	12 (30.0)	12 (30.0)	6 (15.4)	8 (20.0)	11 (27.5)	49 (24.6)
Mild	4 (10.0)	9 (22.5)	4 (10.3)	4 (10.0)	7 (17.5)	28 (14.1)
Moderate	4 (10.0)	2 (5.0)	1 (2.6)	4 (10.0)	3 (7.5)	14 (7.0)
Severe	4 (10.0)	1 (2.5)	1 (2.6)	0	1 (2.5)	7 (3.5)
Cholelithiasis	1 (2.5)	0	0	0	0	1 (0.5)
Mild	1 (2.5)	0	0	0	0	1 (0.5)
Moderate	0	0	0	0	0	0
Severe	0	0	0	0	0	0
Drug-induced liver injury	12 (30.0)	12 (30.0)	6 (15.4)	8 (20.0)	11 (27.5)	49 (24.6)
Mild	4 (10.0)	9 (22.5)	4 (10.3)	4 (10.0)	7 (17.5)	28 (14.1)
Moderate	4 (10.0)	2 (5.0)	1 (2.6)	4 (10.0)	3 (7.5)	14 (7.0)
Severe	4 (10.0)	1 (2.5)	1 (2.6)	0	1 (2.5)	7 (3.5)
**Immune system disorders**	0	2 (5.0)	0	0	0	2 (1.0)

**System Organ Class Preferred Term Severity**	**Control (N=40)**	**CC-11050 (N=40)**	**Everolimus (N=39)**	**Auranofin (N=40)**	**Vitamin D (N=40)**	**Total (N=199)**
Mild	0	2 (5.0)	0	0	0	2 (1.0)
Moderate	0	0	0	0	0	0
Severe	0	0	0	0	0	0
Hypersensitivity	0	2 (5.0)	0	0	0	2 (1.0)
Mild	0	2 (5.0)	0	0	0	2 (1.0)
Moderate	0	0	0	0	0	0
Severe	0	0	0	0	0	0
**Infections and infestations**	6 (15.0)	4 (10.0)	9 (23.1)	9 (22.5)	10 (25.0)	38 (19.1)
Mild	3 (7.5)	1 (2.5)	1 (2.6)	1 (2.5)	3 (7.5)	9 (4.5)
Moderate	3 (7.5)	3 (7.5)	8 (20.5)	6 (15.0)	6 (15.0)	26 (13.1)
Severe	0	0	0	2 (5.0)	1 (2.5)	3 (1.5)
Abscess	0	0	1 (2.6)	0	0	1 (0.5)
Mild	0	0	0	0	0	0
Moderate	0	0	1 (2.6)	0	0	1 (0.5)
Severe	0	0	0	0	0	0
Acute hepatitis B	0	0	0	1 (2.5)	0	1 (0.5)
Mild	0	0	0	0	0	0
Moderate	0	0	0	1 (2.5)	0	1 (0.5)
Severe	0	0	0	0	0	0
Body tinea	0	0	1 (2.6)	0	0	1 (0.5)
Mild	0	0	0	0	0	0
Moderate	0	0	1 (2.6)	0	0	1 (0.5)
Severe	0	0	0	0	0	0
Bone tuberculosis	0	0	0	0	1 (2.5)	1 (0.5)
Mild	0	0	0	0	0	0
Moderate	0	0	0	0	0	0
Severe	0	0	0	0	1 (2.5)	1 (0.5)
Fungal infection	0	0	0	0	1 (2.5)	1 (0.5)
Mild	0	0	0	0	0	0
Moderate	0	0	0	0	1 (2.5)	1 (0.5)
Severe	0	0	0	0	0	0
Fungal skin infection	0	0	0	0	1 (2.5)	1 (0.5)
Mild	0	0	0	0	1 (2.5)	1 (0.5)
Moderate	0	0	0	0	0	0
Severe	0	0	0	0	0	0
Gastroenteritis	0	0	2 (5.1)	3 (7.5)	1 (2.5)	6 (3.0)
Mild	0	0	0	0	1 (2.5)	1 (0.5)
Moderate	0	0	2 (5.1)	2 (5.0)	0	4 (2.0)

System Organ<br>Class Preferred Term<br>Severity	Control (N=40)	CC-11050 (N=40)	Everolimus (N=39)	Auranofin (N=40)	Vitamin D (N=40)	Total (N=199)
Severe	0	0	0	1 (2.5)	0	1 (0.5)
Gingivitis	0	0	0	1 (2.5)	0	1 (0.5)
Mild	0	0	0	0	0	0
Moderate	0	0	0	1 (2.5)	0	1 (0.5)
Severe	0	0	0	0	0	0
Hepatitis B	0	0	0	1 (2.5)	0	1 (0.5)
Mild	0	0	0	0	0	0
Moderate	0	0	0	0	0	0
Severe	0	0	0	1 (2.5)	0	1 (0.5)
Infection	0	0	0	0	1 (2.5)	1 (0.5)
Mild	0	0	0	0	0	0
Moderate	0	0	0	0	1 (2.5)	1 (0.5)
Severe	0	0	0	0	0	0
Lower respiratory tract infection	0	2 (5.0)	2 (5.1)	2 (5.0)	3 (7.5)	9 (4.5)
Mild	0	0	0	0	0	0
Moderate	0	2 (5.0)	2 (5.1)	2 (5.0)	3 (7.5)	9 (4.5)
Severe	0	0	0	0	0	0
Oral candidiasis	0	0	0	0	1 (2.5)	1 (0.5)
Mild	0	0	0	0	0	0
Moderate	0	0	0	0	1 (2.5)	1 (0.5)
Severe	0	0	0	0	0	0
Otitis media	0	0	1 (2.6)	0	0	1 (0.5)
Mild	0	0	0	0	0	0
Moderate	0	0	1 (2.6)	0	0	1 (0.5)
Severe	0	0	0	0	0	0
Rectal abscess	1 (2.5)	0	0	0	0	1 (0.5)
Mild	0	0	0	0	0	0
Moderate	1 (2.5)	0	0	0	0	1 (0.5)
Severe	0	0	0	0	0	0
Respiratory tract infection	1 (2.5)	0	0	0	0	1 (0.5)
Mild	1 (2.5)	0	0	0	0	1 (0.5)
Moderate	0	0	0	0	0	0
Severe	0	0	0	0	0	0
Rhinitis	1 (2.5)	0	0	0	0	1 (0.5)
Mild	0	0	0	0	0	0
Moderate	1 (2.5)	0	0	0	0	1 (0.5)
Severe	0	0	0	0	0	0

System Organ Class Preferred Term Severity	Control (N=40)	CC-11050 (N=40)	Everolimus (N=39)	Auranofin (N=40)	Vitamin D (N=40)	Total (N=199)
Sepsis	0	0	0	1 (2.5)	0	1 (0.5)
Mild	0	0	0	0	0	0
Moderate	0	0	0	0	0	0
Severe	0	0	0	1 (2.5)	0	1 (0.5)
Sexually transmitted disease	1 (2.5)	0	0	0	0	1 (0.5)
Mild	0	0	0	0	0	0
Moderate	1 (2.5)	0	0	0	0	1 (0.5)
Severe	0	0	0	0	0	0
Tinea manuum	0	0	1 (2.6)	0	0	1 (0.5)
Mild	0	0	1 (2.6)	0	0	1 (0.5)
Moderate	0	0	0	0	0	0
Severe	0	0	0	0	0	0
Tinea pedis	0	0	1 (2.6)	0	0	1 (0.5)
Mild	0	0	1 (2.6)	0	0	1 (0.5)
Moderate	0	0	0	0	0	0
Severe	0	0	0	0	0	0
Tonsillitis	0	0	1 (2.6)	0	0	1 (0.5)
Mild	0	0	1 (2.6)	0	0	1 (0.5)
Moderate	0	0	0	0	0	0
Severe	0	0	0	0	0	0
Tooth abscess	0	0	1 (2.6)	0	0	1 (0.5)
Mild	0	0	0	0	0	0
Moderate	0	0	1 (2.6)	0	0	1 (0.5)
Severe	0	0	0	0	0	0
Upper respiratory tract infection	2 (5.0)	1 (2.5)	1 (2.6)	1 (2.5)	1 (2.5)	6 (3.0)
Mild	1 (2.5)	1 (2.5)	0	1 (2.5)	1 (2.5)	4 (2.0)
Moderate	1 (2.5)	0	1 (2.6)	0	0	2 (1.0)
Severe	0	0	0	0	0	0
Urinary tract infection	1 (2.5)	2 (5.0)	0	1 (2.5)	2 (5.0)	6 (3.0)
Mild	1 (2.5)	0	0	0	2 (5.0)	3 (1.5)
Moderate	0	2 (5.0)	0	1 (2.5)	0	3 (1.5)
Severe	0	0	0	0	0	0
**Injury, poisoning and procedural complications**	1 (2.5)	1 (2.5)	1 (2.6)	1 (2.5)	2 (5.0)	6 (3.0)
Mild	0	1 (2.5)	1 (2.6)	1 (2.5)	1 (2.5)	4 (2.0)
Moderate	1 (2.5)	0	0	0	1 (2.5)	2 (1.0)
Severe	0	0	0	0	0	0

System Organ Class Preferred Term Severity	Control (N=40)	CC-11050 (N=40)	Everolimus (N=39)	Auranofin (N=40)	Vitamin D (N=40)	Total (N=199)
Contusion	1 (2.5)	0	0	0	0	1 (0.5)
Mild	0	0	0	0	0	0
Moderate	1 (2.5)	0	0	0	0	1 (0.5)
Severe	0	0	0	0	0	0
Joint injury	0	0	1 (2.6)	0	0	1 (0.5)
Mild	0	0	1 (2.6)	0	0	1 (0.5)
Moderate	0	0	0	0	0	0
Severe	0	0	0	0	0	0
Skin laceration	0	0	0	1 (2.5)	0	1 (0.5)
Mild	0	0	0	1 (2.5)	0	1 (0.5)
Moderate	0	0	0	0	0	0
Severe	0	0	0	0	0	0
Soft tissue injury	0	1 (2.5)	0	0	1 (2.5)	2 (1.0)
Mild	0	1 (2.5)	0	0	0	1 (0.5)
Moderate	0	0	0	0	1 (2.5)	1 (0.5)
Severe	0	0	0	0	0	0
Thermal burn	0	0	0	0	1 (2.5)	1 (0.5)
Mild	0	0	0	0	1 (2.5)	1 (0.5)
Moderate	0	0	0	0	0	0
Severe	0	0	0	0	0	0
**Investigations**	20 (50.0)	18 (45.0)	12 (30.8)	18 (45.0)	21 (52.5)	89 (44.7)
Mild	11 (27.5)	11 (27.5)	8 (20.5)	11 (27.5)	15 (37.5)	56 (28.1)
Moderate	6 (15.0)	5 (12.5)	3 (7.7)	6 (15.0)	5 (12.5)	25 (12.6)
Severe	3 (7.5)	2 (5.0)	1 (2.6)	1 (2.5)	1 (2.5)	8 (4.0)
Alanine aminotransferase increased	0	1 (2.5)	1 (2.6)	0	0	2 (1.0)
Mild	0	0	0	0	0	0
Moderate	0	0	1 (2.6)	0	0	1 (0.5)
Severe	0	1 (2.5)	0	0	0	1 (0.5)
Aspartate aminotransferase increased	10 (25.0)	11 (27.5)	6 (15.4)	8 (20.0)	11 (27.5)	46 (23.1)
Mild	4 (10.0)	9 (22.5)	4 (10.3)	4 (10.0)	7 (17.5)	28 (14.1)
Moderate	4 (10.0)	2 (5.0)	1 (2.6)	4 (10.0)	3 (7.5)	14 (7.0)
Severe	2 (5.0)	0	1 (2.6)	0	1 (2.5)	4 (2.0)
Blood alkaline phosphatase increased	4 (10.0)	4 (10.0)	0	3 (7.5)	6 (15.0)	17 (8.5)
Mild	3 (7.5)	3 (7.5)	0	3 (7.5)	6 (15.0)	15 (7.5)
Moderate	1 (2.5)	1 (2.5)	0	0	0	2 (1.0)
Severe	0	0	0	0	0	0

System Organ Class Preferred Term Severity	Control (N=40)	CC-11050 (N=40)	Everolimus (N=39)	Auranofin (N=40)	Vitamin D (N=40)	Total (N=199)
Blood creatinine increased	2 (5.0)	0	0	0	0	2 (1.0)
Mild	2 (5.0)	0	0	0	0	2 (1.0)
Moderate	0	0	0	0	0	0
Severe	0	0	0	0	0	0
Blood glucose decreased	0	1 (2.5)	3 (7.7)	1 (2.5)	1 (2.5)	6 (3.0)
Mild	0	1 (2.5)	3 (7.7)	1 (2.5)	1 (2.5)	6 (3.0)
Moderate	0	0	0	0	0	0
Severe	0	0	0	0	0	0
Blood glucose increased	0	0	0	0	1 (2.5)	1 (0.5)
Mild	0	0	0	0	1 (2.5)	1 (0.5)
Moderate	0	0	0	0	0	0
Severe	0	0	0	0	0	0
Blood potassium increased	0	0	0	1 (2.5)	0	1 (0.5)
Mild	0	0	0	1 (2.5)	0	1 (0.5)
Moderate	0	0	0	0	0	0
Severe	0	0	0	0	0	0
Blood pressure increased	4 (10.0)	0	3 (7.7)	3 (7.5)	1 (2.5)	11 (5.5)
Mild	3 (7.5)	0	2 (5.1)	3 (7.5)	1 (2.5)	9 (4.5)
Moderate	0	0	1 (2.6)	0	0	1 (0.5)
Severe	1 (2.5)	0	0	0	0	1 (0.5)
Blood uric acid increased	0	1 (2.5)	0	0	0	1 (0.5)
Mild	0	0	0	0	0	0
Moderate	0	0	0	0	0	0
Severe	0	1 (2.5)	0	0	0	1 (0.5)
Breath sounds abnormal	0	0	0	0	1 (2.5)	1 (0.5)
Mild	0	0	0	0	1 (2.5)	1 (0.5)
Moderate	0	0	0	0	0	0
Severe	0	0	0	0	0	0
Electrocardiogram abnormal	1 (2.5)	0	1 (2.6)	0	0	2 (1.0)
Mild	1 (2.5)	0	0	0	0	1 (0.5)
Moderate	0	0	1 (2.6)	0	0	1 (0.5)
Severe	0	0	0	0	0	0
Haemoglobin decreased	2 (5.0)	3 (7.5)	1 (2.6)	1 (2.5)	0	7 (3.5)
Mild	1 (2.5)	2 (5.0)	1 (2.6)	0	0	4 (2.0)
Moderate	1 (2.5)	1 (2.5)	0	1 (2.5)	0	3 (1.5)
Severe	0	0	0	0	0	0
Heart rate increased	1 (2.5)	1 (2.5)	0	1 (2.5)	2 (5.0)	5 (2.5)

**System Organ Class Preferred Term Severity**	**Control (N=40)**	**CC-11050 (N=40)**	**Everolimus (N=39)**	**Auranofin (N=40)**	**Vitamin D (N=40)**	**Total (N=199)**
Mild	1 (2.5)	0	0	1 (2.5)	2 (5.0)	4 (2.0)
Moderate	0	1 (2.5)	0	0	0	1 (0.5)
Severe	0	0	0	0	0	0
Hepatic enzyme increased	2 (5.0)	0	0	0	0	2 (1.0)
Mild	0	0	0	0	0	0
Moderate	1 (2.5)	0	0	0	0	1 (0.5)
Severe	1 (2.5)	0	0	0	0	1 (0.5)
Lymphocyte morphology abnormal	0	1 (2.5)	0	0	0	1 (0.5)
Mild	0	1 (2.5)	0	0	0	1 (0.5)
Moderate	0	0	0	0	0	0
Severe	0	0	0	0	0	0
Neutrophil count decreased	0	0	1 (2.6)	1 (2.5)	1 (2.5)	3 (1.5)
Mild	0	0	1 (2.6)	1 (2.5)	0	2 (1.0)
Moderate	0	0	0	0	1 (2.5)	1 (0.5)
Severe	0	0	0	0	0	0
Oxygen saturation decreased	3 (7.5)	3 (7.5)	4 (10.3)	2 (5.0)	4 (10.0)	16 (8.0)
Mild	3 (7.5)	3 (7.5)	4 (10.3)	2 (5.0)	4 (10.0)	16 (8.0)
Moderate	0	0	0	0	0	0
Severe	0	0	0	0	0	0
Platelet count decreased	0	0	0	1 (2.5)	1 (2.5)	2 (1.0)
Mild	0	0	0	0	1 (2.5)	1 (0.5)
Moderate	0	0	0	1 (2.5)	0	1 (0.5)
Severe	0	0	0	0	0	0
Platelet count increased	0	0	0	1 (2.5)	0	1 (0.5)
Mild	0	0	0	0	0	0
Moderate	0	0	0	1 (2.5)	0	1 (0.5)
Severe	0	0	0	0	0	0
Transaminases abnormal	0	0	0	1 (2.5)	0	1 (0.5)
Mild	0	0	0	0	0	0
Moderate	0	0	0	0	0	0
Severe	0	0	0	1 (2.5)	0	1 (0.5)
Urine analysis abnormal	1 (2.5)	0	0	0	0	1 (0.5)
Mild	1 (2.5)	0	0	0	0	1 (0.5)
Moderate	0	0	0	0	0	0
Severe	0	0	0	0	0	0
Weight decreased	1 (2.5)	0	0	0	1 (2.5)	2 (1.0)

System Organ Class Preferred Term Severity	Control (N=40)	CC-11050 (N=40)	Everolimus (N=39)	Auranofin (N=40)	Vitamin D (N=40)	Total (N=199)
Mild	1 (2.5)	0	0	0	0	1 (0.5)
Moderate	0	0	0	0	1 (2.5)	1 (0.5)
Severe	0	0	0	0	0	0
**Metabolism and nutrition disorders**	3 (7.5)	9 (22.5)	4 (10.3)	7 (17.5)	5 (12.5)	28 (14.1)
Mild	2 (5.0)	6 (15.0)	1 (2.6)	4 (10.0)	4 (10.0)	17 (8.5)
Moderate	1 (2.5)	3 (7.5)	3 (7.7)	2 (5.0)	0	9 (4.5)
Severe	0	0	0	1 (2.5)	1 (2.5)	2 (1.0)
Decreased appetite	0	1 (2.5)	1 (2.6)	2 (5.0)	0	4 (2.0)
Mild	0	1 (2.5)	0	2 (5.0)	0	3 (1.5)
Moderate	0	0	1 (2.6)	0	0	1 (0.5)
Severe	0	0	0	0	0	0
Dehydration	0	0	0	1 (2.5)	0	1 (0.5)
Mild	0	0	0	0	0	0
Moderate	0	0	0	0	0	0
Severe	0	0	0	1 (2.5)	0	1 (0.5)
Diabetes mellitus	0	0	0	1 (2.5)	0	1 (0.5)
Mild	0	0	0	0	0	0
Moderate	0	0	0	1 (2.5)	0	1 (0.5)
Severe	0	0	0	0	0	0
Gout	0	1 (2.5)	0	0	0	1 (0.5)
Mild	0	1 (2.5)	0	0	0	1 (0.5)
Moderate	0	0	0	0	0	0
Severe	0	0	0	0	0	0
Hyperglycaemia	0	1 (2.5)	0	2 (5.0)	1 (2.5)	4 (2.0)
Mild	0	0	0	0	1 (2.5)	1 (0.5)
Moderate	0	1 (2.5)	0	2 (5.0)	0	3 (1.5)
Severe	0	0	0	0	0	0
Hyperkalaemia	0	0	0	1 (2.5)	1 (2.5)	2 (1.0)
Mild	0	0	0	1 (2.5)	1 (2.5)	2 (1.0)
Moderate	0	0	0	0	0	0
Severe	0	0	0	0	0	0
Hypoglycaemia	1 (2.5)	5 (12.5)	2 (5.1)	2 (5.0)	1 (2.5)	11 (5.5)
Mild	1 (2.5)	2 (5.0)	0	2 (5.0)	0	5 (2.5)
Moderate	0	3 (7.5)	2 (5.1)	0	0	5 (2.5)
Severe	0	0	0	0	1 (2.5)	1 (0.5)
Hypokalaemia	2 (5.0)	2 (5.0)	1 (2.6)	1 (2.5)	3 (7.5)	9 (4.5)
Mild	1 (2.5)	2 (5.0)	1 (2.6)	1 (2.5)	3 (7.5)	8 (4.0)

System Organ<br>Class Preferred Term<br>Severity	Control (N=40)	CC-11050 (N=40)	Everolimus (N=39)	Auranofin (N=40)	Vitamin D (N=40)	Total (N=199)
Moderate	1 (2.5)	0	0	0	0	1 (0.5)
Severe	0	0	0	0	0	0
**Musculoskeletal and connective tissue disorders**	11 (27.5)	15 (37.5)	8 (20.5)	11 (27.5)	15 (37.5)	60 (30.2)
Mild	5 (12.5)	8 (20.0)	4 (10.3)	4 (10.0)	11 (27.5)	32 (16.1)
Moderate	6 (15.0)	7 (17.5)	4 (10.3)	7 (17.5)	4 (10.0)	28 (14.1)
Severe	0	0	0	0	0	0
Arthralgia	6 (15.0)	8 (20.0)	4 (10.3)	8 (20.0)	9 (22.5)	35 (17.6)
Mild	4 (10.0)	4 (10.0)	2 (5.1)	2 (5.0)	6 (15.0)	18 (9.0)
Moderate	2 (5.0)	4 (10.0)	2 (5.1)	6 (15.0)	3 (7.5)	17 (8.5)
Severe	0	0	0	0	0	0
Arthritis reactive	0	1 (2.5)	1 (2.6)	0	0	2 (1.0)
Mild	0	0	0	0	0	0
Moderate	0	1 (2.5)	1 (2.6)	0	0	2 (1.0)
Severe	0	0	0	0	0	0
Back pain	4 (10.0)	1 (2.5)	1 (2.6)	4 (10.0)	8 (20.0)	18 (9.0)
Mild	2 (5.0)	1 (2.5)	1 (2.6)	2 (5.0)	6 (15.0)	12 (6.0)
Moderate	2 (5.0)	0	0	2 (5.0)	2 (5.0)	6 (3.0)
Severe	0	0	0	0	0	0
Bone pain	0	1 (2.5)	0	0	0	1 (0.5)
Mild	0	0	0	0	0	0
Moderate	0	1 (2.5)	0	0	0	1 (0.5)
Severe	0	0	0	0	0	0
Costochondritis	0	0	1 (2.6)	0	0	1 (0.5)
Mild	0	0	1 (2.6)	0	0	1 (0.5)
Moderate	0	0	0	0	0	0
Severe	0	0	0	0	0	0
Flank pain	0	1 (2.5)	1 (2.6)	0	0	2 (1.0)
Mild	0	1 (2.5)	0	0	0	1 (0.5)
Moderate	0	0	1 (2.6)	0	0	1 (0.5)
Severe	0	0	0	0	0	0
Joint swelling	1 (2.5)	2 (5.0)	0	0	0	3 (1.5)
Mild	1 (2.5)	1 (2.5)	0	0	0	2 (1.0)
Moderate	0	1 (2.5)	0	0	0	1 (0.5)
Severe	0	0	0	0	0	0
Muscle spasms	1 (2.5)	1 (2.5)	0	0	1 (2.5)	3 (1.5)
Mild	0	0	0	0	1 (2.5)	1 (0.5)
Moderate	1 (2.5)	1 (2.5)	0	0	0	2 (1.0)

System Organ Class Preferred Term Severity	Control (N=40)	CC-11050 (N=40)	Everolimus (N=39)	Auranofin (N=40)	Vitamin D (N=40)	Total (N=199)
Severe	0	0	0	0	0	0
Myalgia	0	2 (5.0)	0	0	0	2 (1.0)
Mild	0	2 (5.0)	0	0	0	2 (1.0)
Moderate	0	0	0	0	0	0
Severe	0	0	0	0	0	0
Neck pain	0	0	0	0	1 (2.5)	1 (0.5)
Mild	0	0	0	0	1 (2.5)	1 (0.5)
Moderate	0	0	0	0	0	0
Severe	0	0	0	0	0	0
Osteoarthritis	0	0	0	1 (2.5)	0	1 (0.5)
Mild	0	0	0	0	0	0
Moderate	0	0	0	1 (2.5)	0	1 (0.5)
Severe	0	0	0	0	0	0
Pain in extremity	1 (2.5)	2 (5.0)	1 (2.6)	2 (5.0)	0	6 (3.0)
Mild	0	2 (5.0)	1 (2.6)	1 (2.5)	0	4 (2.0)
Moderate	1 (2.5)	0	0	1 (2.5)	0	2 (1.0)
Severe	0	0	0	0	0	0
Pain in jaw	0	1 (2.5)	0	0	0	1 (0.5)
Mild	0	1 (2.5)	0	0	0	1 (0.5)
Moderate	0	0	0	0	0	0
Severe	0	0	0	0	0	0
**Nervous system disorders**	2 (5.0)	8 (20.0)	0	3 (7.5)	6 (15.0)	19 (9.5)
Mild	2 (5.0)	5 (12.5)	0	1 (2.5)	4 (10.0)	12 (6.0)
Moderate	0	3 (7.5)	0	1 (2.5)	2 (5.0)	6 (3.0)
Severe	0	0	0	1 (2.5)	0	1 (0.5)
Dizziness	0	0	0	1 (2.5)	0	1 (0.5)
Mild	0	0	0	0	0	0
Moderate	0	0	0	1 (2.5)	0	1 (0.5)
Severe	0	0	0	0	0	0
Headache	2 (5.0)	3 (7.5)	0	0	1 (2.5)	6 (3.0)
Mild	2 (5.0)	2 (5.0)	0	0	0	4 (2.0)
Moderate	0	1 (2.5)	0	0	1 (2.5)	2 (1.0)
Severe	0	0	0	0	0	0
Neuropathy peripheral	0	5 (12.5)	0	2 (5.0)	5 (12.5)	12 (6.0)
Mild	0	3 (7.5)	0	1 (2.5)	4 (10.0)	8 (4.0)
Moderate	0	2 (5.0)	0	1 (2.5)	1 (2.5)	4 (2.0)
Severe	0	0	0	0	0	0
Syncope	0	0	0	1 (2.5)	0	1 (0.5)

**System Organ Class Preferred Term Severity**	**Control (N=40)**	**CC-11050 (N=40)**	**Everolimus (N=39)**	**Auranofin (N=40)**	**Vitamin D (N=40)**	**Total (N=199)**
Mild	0	0	0	0	0	0
Moderate	0	0	0	0	0	0
Severe	0	0	0	1 (2.5)	0	1 (0.5)
**Psychiatric disorders**	1 (2.5)	0	2 (5.1)	2 (5.0)	0	5 (2.5)
Mild	0	0	1 (2.6)	2 (5.0)	0	3 (1.5)
Moderate	1 (2.5)	0	1 (2.6)	0	0	2 (1.0)
Severe	0	0	0	0	0	0
Insomnia	1 (2.5)	0	1 (2.6)	2 (5.0)	0	4 (2.0)
Mild	0	0	1 (2.6)	2 (5.0)	0	3 (1.5)
Moderate	1 (2.5)	0	0	0	0	1 (0.5)
Severe	0	0	0	0	0	0
Psychotic disorder	0	0	1 (2.6)	0	0	1 (0.5)
Mild	0	0	0	0	0	0
Moderate	0	0	1 (2.6)	0	0	1 (0.5)
Severe	0	0	0	0	0	0
**Renal and urinary disorders**	0	0	1 (2.6)	1 (2.5)	1 (2.5)	3 (1.5)
Mild	0	0	0	1 (2.5)	1 (2.5)	2 (1.0)
Moderate	0	0	1 (2.6)	0	0	1 (0.5)
Severe	0	0	0	0	0	0
Dysuria	0	0	1 (2.6)	1 (2.5)	0	2 (1.0)
Mild	0	0	0	1 (2.5)	0	1 (0.5)
Moderate	0	0	1 (2.6)	0	0	1 (0.5)
Severe	0	0	0	0	0	0
Micturition urgency	0	0	0	0	1 (2.5)	1 (0.5)
Mild	0	0	0	0	1 (2.5)	1 (0.5)
Moderate	0	0	0	0	0	0
Severe	0	0	0	0	0	0
**Reproductive system and breast disorders**	2 (5.0)	3 (7.5)	1 (2.6)	1 (2.5)	3 (7.5)	10 (5.0)
Mild	2 (5.0)	2 (5.0)	1 (2.6)	0	1 (2.5)	6 (3.0)
Moderate	0	1 (2.5)	0	1 (2.5)	2 (5.0)	4 (2.0)
Severe	0	0	0	0	0	0
Dysfunctional uterine bleeding	0	0	0	0	1 (2.5)	1 (0.5)
Mild	0	0	0	0	0	0
Moderate	0	0	0	0	1 (2.5)	1 (0.5)
Severe	0	0	0	0	0	0
Gynaecomastia	0	0	0	0	1 (2.5)	1 (0.5)
Mild	0	0	0	0	0	0

System Organ<br>Class Preferred Term<br>Severity	Control (N=40)	CC-11050 (N=40)	Everolimus (N=39)	Auranofin (N=40)	Vitamin D (N=40)	Total (N=199)
Moderate	0	0	0	0	1 (2.5)	1 (0.5)
Severe	0	0	0	0	0	0
Menorrhagia	0	1 (2.5)	0	0	0	1 (0.5)
Mild	0	1 (2.5)	0	0	0	1 (0.5)
Moderate	0	0	0	0	0	0
Severe	0	0	0	0	0	0
Metrorrhagia	0	1 (2.5)	0	0	0	1 (0.5)
Mild	0	1 (2.5)	0	0	0	1 (0.5)
Moderate	0	0	0	0	0	0
Severe	0	0	0	0	0	0
Pelvic fluid collection	0	0	1 (2.6)	0	0	1 (0.5)
Mild	0	0	1 (2.6)	0	0	1 (0.5)
Moderate	0	0	0	0	0	0
Severe	0	0	0	0	0	0
Prostatic calcification	1 (2.5)	0	0	0	0	1 (0.5)
Mild	1 (2.5)	0	0	0	0	1 (0.5)
Moderate	0	0	0	0	0	0
Severe	0	0	0	0	0	0
Scrotal mass	0	0	0	1 (2.5)	0	1 (0.5)
Mild	0	0	0	0	0	0
Moderate	0	0	0	1 (2.5)	0	1 (0.5)
Severe	0	0	0	0	0	0
Vaginal discharge	0	1 (2.5)	0	0	0	1 (0.5)
Mild	0	0	0	0	0	0
Moderate	0	1 (2.5)	0	0	0	1 (0.5)
Severe	0	0	0	0	0	0
Vaginal haemorrhage	1 (2.5)	1 (2.5)	0	0	1 (2.5)	3 (1.5)
Mild	1 (2.5)	1 (2.5)	0	0	1 (2.5)	3 (1.5)
Moderate	0	0	0	0	0	0
Severe	0	0	0	0	0	0
**Respiratory, thoracic and mediastinal disorders**	12 (30.0)	8 (20.0)	8 (20.5)	10 (25.0)	10 (25.0)	48 (24.1)
Mild	9 (22.5)	8 (20.0)	7 (17.9)	7 (17.5)	8 (20.0)	39 (19.6)
Moderate	3 (7.5)	0	1 (2.6)	2 (5.0)	2 (5.0)	8 (4.0)
Severe	0	0	0	1 (2.5)	0	1 (0.5)
Chest pain	10 (25.0)	3 (7.5)	2 (5.1)	4 (10.0)	6 (15.0)	25 (12.6)
Mild	7 (17.5)	3 (7.5)	2 (5.1)	3 (7.5)	4 (10.0)	19 (9.5)
Moderate	3 (7.5)	0	0	1 (2.5)	2 (5.0)	6 (3.0)

System Organ<br>Class Preferred Term<br>Severity	Control (N=40)	CC-11050 (N=40)	Everolimus (N=39)	Auranofin (N=40)	Vitamin D (N=40)	Total (N=199)
Severe	0	0	0	0	0	0
Cough	2 (5.0)	0	2 (5.1)	2 (5.0)	1 (2.5)	7 (3.5)
Mild	2 (5.0)	0	1 (2.6)	1 (2.5)	1 (2.5)	5 (2.5)
Moderate	0	0	1 (2.6)	1 (2.5)	0	2 (1.0)
Severe	0	0	0	0	0	0
Dyspnoea	0	1 (2.5)	0	1 (2.5)	0	2 (1.0)
Mild	0	1 (2.5)	0	0	0	1 (0.5)
Moderate	0	0	0	1 (2.5)	0	1 (0.5)
Severe	0	0	0	0	0	0
Epistaxis	0	0	0	1 (2.5)	1 (2.5)	2 (1.0)
Mild	0	0	0	1 (2.5)	1 (2.5)	2 (1.0)
Moderate	0	0	0	0	0	0
Severe	0	0	0	0	0	0
Haemoptysis	2 (5.0)	1 (2.5)	1 (2.6)	1 (2.5)	3 (7.5)	8 (4.0)
Mild	2 (5.0)	1 (2.5)	1 (2.6)	1 (2.5)	3 (7.5)	8 (4.0)
Moderate	0	0	0	0	0	0
Severe	0	0	0	0	0	0
Nasal congestion	0	1 (2.5)	0	1 (2.5)	0	2 (1.0)
Mild	0	1 (2.5)	0	1 (2.5)	0	2 (1.0)
Moderate	0	0	0	0	0	0
Severe	0	0	0	0	0	0
Productive cough	0	0	0	1 (2.5)	0	1 (0.5)
Mild	0	0	0	1 (2.5)	0	1 (0.5)
Moderate	0	0	0	0	0	0
Severe	0	0	0	0	0	0
Rales	1 (2.5)	1 (2.5)	2 (5.1)	0	0	4 (2.0)
Mild	1 (2.5)	1 (2.5)	2 (5.1)	0	0	4 (2.0)
Moderate	0	0	0	0	0	0
Severe	0	0	0	0	0	0
Respiration abnormal	0	1 (2.5)	0	0	0	1 (0.5)
Mild	0	1 (2.5)	0	0	0	1 (0.5)
Moderate	0	0	0	0	0	0
Severe	0	0	0	0	0	0
Respiratory failure	0	0	0	1 (2.5)	0	1 (0.5)
Mild	0	0	0	0	0	0
Moderate	0	0	0	0	0	0
Severe	0	0	0	1 (2.5)	0	1 (0.5)
Rhinorrhoea	2 (5.0)	3 (7.5)	1 (2.6)	1 (2.5)	1 (2.5)	8 (4.0)

System Organ Class Preferred Term Severity	Control (N=40)	CC-11050 (N=40)	Everolimus (N=39)	Auranofin (N=40)	Vitamin D (N=40)	Total (N=199)
Mild	2 (5.0)	3 (7.5)	1 (2.6)	1 (2.5)	1 (2.5)	8 (4.0)
Moderate	0	0	0	0	0	0
Severe	0	0	0	0	0	0
Tachypnoea	0	0	1 (2.6)	0	1 (2.5)	2 (1.0)
Mild	0	0	1 (2.6)	0	1 (2.5)	2 (1.0)
Moderate	0	0	0	0	0	0
Severe	0	0	0	0	0	0
Wheezing	3 (7.5)	1 (2.5)	0	1 (2.5)	1 (2.5)	6 (3.0)
Mild	3 (7.5)	1 (2.5)	0	1 (2.5)	1 (2.5)	6 (3.0)
Moderate	0	0	0	0	0	0
Severe	0	0	0	0	0	0
**Skin and subcutaneous tissue disorders**	6 (15.0)	7 (17.5)	9 (23.1)	6 (15.0)	7 (17.5)	35 (17.6)
Mild	4 (10.0)	5 (12.5)	7 (17.9)	5 (12.5)	6 (15.0)	27 (13.6)
Moderate	2 (5.0)	2 (5.0)	2 (5.1)	1 (2.5)	1 (2.5)	8 (4.0)
Severe	0	0	0	0	0	0
Acne	0	1 (2.5)	1 (2.6)	0	1 (2.5)	3 (1.5)
Mild	0	1 (2.5)	1 (2.6)	0	1 (2.5)	3 (1.5)
Moderate	0	0	0	0	0	0
Severe	0	0	0	0	0	0
Dry skin	0	0	1 (2.6)	0	0	1 (0.5)
Mild	0	0	1 (2.6)	0	0	1 (0.5)
Moderate	0	0	0	0	0	0
Severe	0	0	0	0	0	0
Eczema	0	1 (2.5)	0	0	0	1 (0.5)
Mild	0	0	0	0	0	0
Moderate	0	1 (2.5)	0	0	0	1 (0.5)
Severe	0	0	0	0	0	0
Night sweats	1 (2.5)	1 (2.5)	1 (2.6)	1 (2.5)	0	4 (2.0)
Mild	1 (2.5)	1 (2.5)	1 (2.6)	1 (2.5)	0	4 (2.0)
Moderate	0	0	0	0	0	0
Severe	0	0	0	0	0	0
Pruritus	3 (7.5)	0	4 (10.3)	4 (10.0)	4 (10.0)	15 (7.5)
Mild	2 (5.0)	0	3 (7.7)	3 (7.5)	4 (10.0)	12 (6.0)
Moderate	1 (2.5)	0	1 (2.6)	1 (2.5)	0	3 (1.5)
Severe	0	0	0	0	0	0
Rash	2 (5.0)	5 (12.5)	3 (7.7)	1 (2.5)	3 (7.5)	14 (7.0)
Mild	1 (2.5)	4 (10.0)	2 (5.1)	1 (2.5)	2 (5.0)	10 (5.0)

System Organ Class Preferred Term Severity	Control (N=40)	CC-11050 (N=40)	Everolimus (N=39)	Auranofin (N=40)	Vitamin D (N=40)	Total (N=199)
Moderate	1 (2.5)	1 (2.5)	1 (2.6)	0	1 (2.5)	4 (2.0)
Severe	0	0	0	0	0	0
**Social circumstances**	0	1 (2.5)	0	0	1 (2.5)	2 (1.0)
Mild	0	1 (2.5)	0	0	0	1 (0.5)
Moderate	0	0	0	0	1 (2.5)	1 (0.5)
Severe	0	0	0	0	0	0
Physical assault	0	1 (2.5)	0	0	1 (2.5)	2 (1.0)
Mild	0	1 (2.5)	0	0	0	1 (0.5)
Moderate	0	0	0	0	1 (2.5)	1 (0.5)
Severe	0	0	0	0	0	0
**Surgical and medical procedures**	0	0	0	1 (2.5)	0	1 (0.5)
Mild	0	0	0	0	0	0
Moderate	0	0	0	1 (2.5)	0	1 (0.5)
Severe	0	0	0	0	0	0
Hospitalisation	0	0	0	1 (2.5)	0	1 (0.5)
Mild	0	0	0	0	0	0
Moderate	0	0	0	1 (2.5)	0	1 (0.5)
Severe	0	0	0	0	0	0
**Vascular disorders**	2 (5.0)	2 (5.0)	2 (5.1)	5 (12.5)	2 (5.0)	13 (6.5)
Mild	1 (2.5)	2 (5.0)	2 (5.1)	3 (7.5)	1 (2.5)	9 (4.5)
Moderate	1 (2.5)	0	0	1 (2.5)	1 (2.5)	3 (1.5)
Severe	0	0	0	1 (2.5)	0	1 (0.5)
Hot flush	0	1 (2.5)	0	0	0	1 (0.5)
Mild	0	1 (2.5)	0	0	0	1 (0.5)
Moderate	0	0	0	0	0	0
Severe	0	0	0	0	0	0
Hypertension	2 (5.0)	1 (2.5)	2 (5.1)	4 (10.0)	2 (5.0)	11 (5.5)
Mild	1 (2.5)	1 (2.5)	2 (5.1)	2 (5.0)	1 (2.5)	7 (3.5)
Moderate	1 (2.5)	0	0	1 (2.5)	1 (2.5)	3 (1.5)
Severe	0	0	0	1 (2.5)	0	1 (0.5)
Hypotension	0	0	0	1 (2.5)	0	1 (0.5)
Mild	0	0	0	1 (2.5)	0	1 (0.5)
Moderate	0	0	0	0	0	0
Severe	0	0	0	0	0	0

Table S2. Hazard ratio (HR) for stable culture conversion in the mITT population relative to control.

**Treatment arm**	**Unadjusted**			**Adjusted[a]**		
	**HR**	**95% CI**	**P**	**HR**	**95% CI**	**P**
CC-11050	1.34	0.86;2.10	0.20	1.32	0.84;2.07	0.23
Everolimus	1.22	0.78;1.92	0.39	1.32	0.83;2.08	0.24
Auranofin	1.12	0.71;1.75	0.63	1.17	0.74;1.84	0.50
Ergocalciferol	1.07	0.69;1.66	0.77	1.00	0.65;1.57	0.97

Stable culture conversion was determined using both liquid and solid cultures. [a]Analysis was adjusted for differences at baseline in MGIT TTP.

Table S3. Hazard ratio (HR) for stable culture conversion in the PP population relative to control, using liquid cultures only.

**Treatment arm**	**Unadjusted**			**Adjusted[a]**		
	**HR**	**95% CI**	**P**	**HR**	**95% CI**	**P**
CC-11050	1.10	0.70; 1.73	0.68	0.94	0.60; 1.49	0.80
Everolimus	1.13	0.71; 1.78	0.62	1.13	0.71; 1.79	0.60
Auranofin	1.21	0.77; 1.91	0.41	1.28	0.81; 2.01	0.30
Ergocalciferol	0.96	0.61; 1.50	0.84	0.90	0.57; 1.42	0.67

PP=per protocol; CI=confidence interval. [a]HR was adjusted for differences in MGIT TTP at baseline.

Table S4. Spirometry outcomes in the mITT population.

Treatment	At 2 months						At 6 months					
Arm	Unadjusted			Adjusted[a]			Unadjusted			Adjusted[a]		
**FEV1 (%)**	**Mean**	**95% CI**	**P**	**Mean**	**95% CI**	**P**	**Mean**	**95% CI**	**P**	**Mean**	**95% CI**	**P**
CC-11050	3.54	-5.57;12.7	0.44	1.63	-5.20;8.46	0.64	8.40	-0.22;17.0	0.06	6.30	0.09;12.5	0.05
Everolimus	4.81	-4.43;14.1	0.31	-0.13	-7.06;6.80	0.97	11.1	2.40;19.9	0.01	6.34	0.04;12.6	0.05
Auranofin	-5.72	-14.8;3.33	0.21	-5.06	-11.8;1.68	0.14	-3.70	-12.4;4.98	0.40	-2.79	-9.00;3.42	0.38
Ergocalciferol	-0.49	-9.54;8.56	0.92	-2.88	-9.63;3.87	0.40	2.61	-6.01;11.2	0.55	0.66	-5.52;6.83	0.83
**FVC (L)**						**P**						
CC-11050	-0.04	-0.41;0.33	0.82	0.07	-0.18;0.32	0.59	0.07	-0.30;0.43	0.71	0.18	-0.08;0.45	0.18
Everolimus	0.08	-0.29;0.45	0.68	0.09	-0.16;0.34	0.49	0.08	-0.29;0.45	0.68	0.10	-0.17;0.37	0.47
Auranofin	-0.11	-0.48;0.25	0.54	-0.03	-0.27;0.22	0.82	-0.12	-0.49;0.25	0.52	-0.02	-0.29;0.25	0.89
Ergocalciferol	-0.14	-0.50;0.23	0.46	-0.06	-0.30;0.19	0.65	-0.18	-0.55;0.19	0.33	-0.04	-0.31;0.23	0.77

Mean values indicate difference from controls. [a]FEV1 analyses were adjusted for baseline differences in FEV1. FVC analyses were adjusted for baseline differences in FVC.

Table S5. Spirometry outcomes in the per protocol (PP) population, including an adjustment for site.

Treatment	At 2 months						At 6 months					
**Arm**	**Unadjusted**			**Adjusted[a]**			**Unadjusted**			**Adjusted[a]**		
**FEV1 (%)**	**Mean**	**95% CI**	**P**	**Mean**	**95% CI**	**P**	**Mean**	**95% CI**	**P**	**Mean**	**95% CI**	**P**
CC-11050	3.54	-5.57;12.7	0.44	1.68	-5.19;8.55	0.63	8.40	-0.24;17.0	0.06	6.32	0.12;12.5	0.05
Everolimus	5.63	-3.68;14.9	0.23	0.52	-6.51;7.56	0.88	11.7	2.84;20.5	0.01	6.70	0.36;13.0	0.04
Auranofin	-6.17	-15.3;2.95	0.18	-5.00	-11.8;1.83	0.15	-3.70	-12.4;5.00	0.40	-2.56	-8.77;3.64	0.42
Ergocalciferol	-0.64	-9.76;8.48	0.89	-2.71	-9.55;4.12	0.43	2.35	-6.34;11.1	0.59	0.66	-5.55;6.87	0.83
**FVC (L)**												
CC-11050	-0.04	-0.41;0.32	0.82	0.07	-0.18;0.32	0.58	0.07	-0.30;0.43	0.71	0.18	-0.09;0.45	0.19
Everolimus	0.11	-0.26;0.48	0.57	0.11	-0.14;0.37	0.37	0.09	-0.28;0.47	0.62	0.11	-0.17;0.38	0.44
Auranofin	-0.14	-0.50;0.23	0.45	-0.03	-0.28;0.22	0.81	-0.12	-0.49;0.25	0.52	-0.01	-0.28;0.26	0.93
Ergocalciferol	-0.15	-0.51;0.22	0.43	-0.04	-0.29;0.21	0.74	-0.20	-0.57;0.17	0.29	-0.04	-0.31;0.23	0.78

Mean values indicate difference from controls. [a]FEV1 analyses were adjusted for baseline differences in FEV1 and site. FVC analyses were adjusted for baseline differences in FVC and site.

Table S6. Effects on FEV1 at 6 months considering only ATS/ERS grade categories A-D.

Treatment arm	N	Mean FEV1	Unadjusted			Adjusted[a]		
			Mean difference from control	95% CI	P	Mean difference from control	95% CI	P
Control	37	68.9%	0				0	
CC-11050	36	77.8%	8.80	-.25; 17.86	0.06	7.08	0.71; 13.46	0.03
Everolimus	30	80.1%	11.15	1.65; 20.65	0.02	6.67	-.01; 13.35	0.05
Auranofin	32	65.8%	-3.13	-12.47; 6.20	0.51	-3.31	-9.84; 3.21	0.32
Ergocalciferol	35	72.5%	3.57	-5.55; 12.69	0.44	2.06	-4.32; 8.44	0.52

The primary analysis of FEV1 considered American Thoracic Society/European Respiratory Society grades A-E acceptable, and U-F unacceptable. Using that threshold, all but 1 patient could perform spirometry acceptably for FEV1 on day 1. However, 24.2% of these evaluations were grade E, the lowest acceptable grade, due to a difference between the 2 highest attempts >0.200L. This proportion decreased progressively to 9.6% at day 180, presumably as an effect of treatment. To assess the potential influence of uncertainty regarding measurement of FEV1 on study findings, we performed a post-hoc analysis in which only grades A-D were considered (above). The analysis examined differences from control at month 6, before and after adjustment for differences in day 1 FEV1 values. These results confirmed the findings of the primary analysis. [a]Analyses were adjusted for baseline differences in FEV1.

Table S7. Effects on FEV1 over time in the mITT population using a random effects model.

**Treatment arm**	**Difference from control for the slope parameter**		
	Coefficient	95% CI	P-value
CC-11050	.030	.004; .055	0.021
Everolimus	.022	-.003; .048	0.083
Auranofin	.000	-.025; .025	0.999
Ergocalciferol	.016	-.009; .041	0.218

We fit a random effects model for the repeated measurements of FEV1% linearly associated with study day to day 180. It included an interaction between study arm and day. The significance level of the overall interaction was P=0.07. Table S7 shows the difference from control in the slope parameter according to treatment arm. A model using ln(FEV1%) performed similarly (P=.04), as did others that included a quadratic term for day (P=.07 to .08). These findings are consistent with the analysis reported in table 3.

Table S8. Criteria for classifying radiographic extent of disease in tuberculosis.

Adapted from Diagnostic standards and classification of tuberculosis, National Tuberculosis Association, 1940

Slight to moderate density lesions total volume	see note 1 below	☒		
	between note 1 and 1 lung		☐	
	>1 lung			☐
Dense or confluent lesions total volume	0	☒		
	between 0 and 1/3 lung		☐	
	>1/3 lung			☐
Cavity total diameter (see note 3 below)	0 cm	☒		
	between 0 and 4 cm		☐	
	≥4 cm			☐
Overall extent of disease	minimal	☒		
	moderately advanced		☐	
	far advanced			☐

Notes:

1. To qualify as minimal disease, lesions must be of slight to moderate density only, and must occupy no more than the volume defined by the 2nd chondrosternal junction and the spine of the fourth or the body of the fifth thoracic vertebra on one side (see below)
2. Please note that although total volume may be compared to that of 1 lung, it may be distributed across both lungs
3. If conventional full size films are used, total cavity diameter may be measured directly. For other image types, a scaling factor may be applied if one is provided. Otherwise, the width of the anterior portion of the 3rd or 4th rib may be used to approximate 1 cm.
4. All checks must be in white for minimal disease
5. Any checks in black result in far advanced disease
6. Everything else is moderately advanced disease

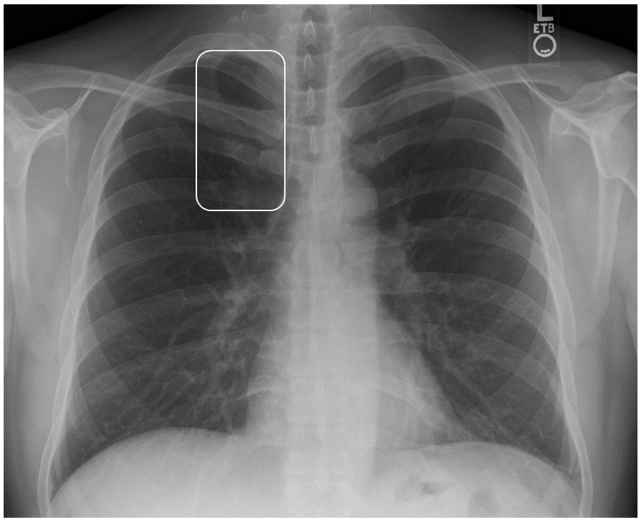

Natural history of pulmonary tuberculosis in the pre-chemotherapy era, according to radiographic extent of disease at diagnosis. From Alling DW and Bosworth EB. The after-history of pulmonary tuberculosis. ARRD 81:839-849, 1960.

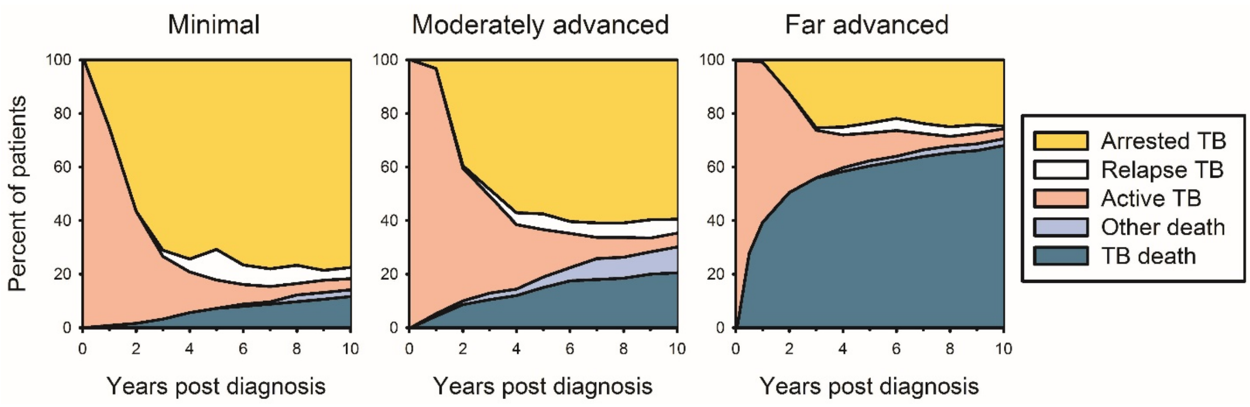

To be classified as “arrested TB”, constitutional symptoms must be absent, sputum if any, must be … microscopically negative for tubercle bacilli, lesions stationary and apparently healed according to X-ray examination with no evidence of pulmonary cavity. These conditions shall have existed for a period of six months, during the last two of which the patient has been taking one hour's walking exercise twice daily or its equivalent. From Diagnostic standards and classification of tuberculosis, National Tuberculosis Association, 1940.
